# *Helicobacter pylori* glycan biosynthesis modulates host immune cell recognition and response

**DOI:** 10.3389/fcimb.2024.1377077

**Published:** 2024-03-20

**Authors:** Katharine A. Barrett, Francis Jacob Kassama, William Surks, Andrew J. Mulholland, Karen D. Moulton, Danielle H. Dube

**Affiliations:** Department of Chemistry & Biochemistry, Bowdoin College, Brunswick, ME, United States

**Keywords:** glycan, immunology, glycosylation mutant, phase variation, *Helicobacter pylori*, metabolic labeling

## Abstract

**Introduction:**

The pathogenic bacterium *Helicobacter pylori* has evolved glycan-mediated mechanisms to evade host immune defenses. This study tests the hypothesis that genetic disruption of *H. pylori* glycan biosynthesis alters immune recognition and response by human gastric epithelial cells and monocyte-derived dendritic cells.

**Methods:**

To test this hypothesis, human cell lines were challenged with wildtype *H. pylori* alongside an array of *H. pylori* glycosylation mutants. The relative levels of immune response were measured via immature dendritic cell maturation and cytokine secretion.

**Results:**

Our findings indicate that disruption of lipopolysaccharide biosynthesis diminishes gastric cytokine production, without disrupting dendritic cell recognition and activation. In contrast, variable immune responses were observed in protein glycosylation mutants which prompted us to test the hypothesis that phase variation plays a role in regulating bacterial cell surface glycosylation and subsequent immune recognition. Lewis antigen presentation does not correlate with extent of immune response, while the extent of lipopolysaccharide O-antigen elaboration does.

**Discussion:**

The outcomes of this study demonstrate that *H. pylori* glycans modulate the host immune response. This work provides a foundation to pursue immune-based tailoring of bacterial glycans towards modulating immunogenicity of microbial pathogens.

## 1 Introduction

Antibiotics are losing their therapeutic efficacy due to the evolution and spread of resistance mechanisms in bacterial pathogens (Thung et al., 2016). The gram-negative gastric bacterium *Helicobacter pylori* is on the World Health Organization’s list of antibiotic-resistant bacteria of highest priority for new antibiotic development (Dong and Graham, 2017; Tshibangu-Kabamba and Yamaoka, 2021). *H. pylori* is an opportunistic, gram-negative bacteria present in the gastrointestinal tract of roughly 50% of people worldwide (Dunne et al., 2014). Despite its relative ubiquity, *H. pylori* only causes severe pathologies in roughly 15% of cases, in which the infection may transform quickly from digestive issues and energy depletion to peptic ulcer disease and gastric carcinomas (Suerbaum and Michetti, 2002; Chen et al., 2013; Kim, 2016; Bravo et al., 2018; Malfertheiner et al., 2023). Recent studies suggest links between *H. pylori* pathogenesis and extra-gastric diseases, including cardiovascular, neurologic, and dermatologic conditions (Gravina et al., 2018). Moreover, low-income communities are at higher risk for infection, as transmission is likely based in contaminated drinking water and ill-resourced sanitation systems (Kim, 2016; Lim et al., 2013; Stefano et al., 2018). *H. pylori* can persistently colonize the human stomach for decades despite development of an immune response. Further, recurrence of infection after initial eradication due to ineffective treatment, dormant state changes, or reinfection poses a threat within the current climate of increased antibiotic resistance (Cellini et al., 2008; Moya and Crissinger, 2012; Hu et al., 2017; Reshetnyak and Reshetnyak, 2017; Di Fermo et al., 2023). These factors point to an urgent need to identify novel approaches to mitigate *H. pylori* pathogenesis.

*H. pylori’s* pathogenesis in some environments and dormancy in others suggests it may be possible to modulate the deleterious consequences of infection. In particular, extensive research demonstrates that localized host immune response to *H. pylori* is directly correlated with patient outcome. Upregulation of pro-inflammatory signaling in *H. pylori* infection, such as the secretion of interleukin-8 and other cytokines (El Filaly et al., 2023), drives the chronic inflammation underpinning gastritis and increases risk for peptic ulcer disease and gastric carcinomas (Robinson et al., 2007; Lamb and Chen, 2013; White and Winter, 2015). Thus, tailoring host immune response has the potential to alter the course of infection, promoting eradication while minimizing downstream consequences of chronic inflammation.

Several factors have been identified that contribute to chronic inflammation and carcinogenesis (Baj et al., 2020). *H. pylori* produces proteases to degrade mucins protecting epithelial cells and trigger cytokine production by the immune system (Byrd et al., 2000; Yoshimura et al., 2002; Posselt et al., 2017). Moreover, *H. pylori* secretes urease to yield ammonia and neutralize the acidic stomach, and this protein drives inflammation by activating immune cells (Olivera-Severo et al., 2017). Data indicate that *H. pylori* expression levels of certain adhesins, BabA and SabA (Doohan et al., 2021), toxins, VacA (Cover and Blanke, 2005), and other virulence-associated proteins, OipA and DupA, increase pathogenicity of infection (Yamaoka et al., 2002; Lu et al., 2005). Notoriously, upregulation of *H. pylori* CagA protein and the type IV secretion system encoded by the *cagA* pathogenicity island leads to injection of CagA, peptidoglycan, and other molecules into host cells, greatly increasing inflammation (Vannini et al., 2014). Gastric inflammation driven by CagA is so severe that it is correlated with gastric carcinogenesis and CagA is considered oncogenic (Tanaka et al., 2010; Jiménez-Soto and Haas, 2016). However, the correlation between these factors and patient pathology is not always linear; other determinants such as host genotype, age of infection, and environmental factors additionally complicate the distribution of inflammation, gastritis, and subsequent pathologies (El-Omar et al., 2003; MaChado et al., 2003; Lu et al., 2005). Regardless of its source, inflammation levels and the unique immune response induced by an *H. pylori* infection determine disease outcome (Robinson et al., 2007; White and Winter, 2015; Gobert and Wilson, 2022). Further investigating pathways by which *H. pylori* evades or stimulates human immune recognition will promote the development of immune-based treatments to bacterial infection.

The glycocalyx, which serves as the interface for host-bacterial interactions, may be key to understanding and ultimately modulating *H. pylori*’s immunogenicity. *H. pylori* adorns its cell envelope with carbohydrates termed glycans (Tra and Dube, 2014). Broadly, bacterial glycans play roles in cell adhesion, host mimicry, and immune evasion (Tra and Dube, 2014; Prado Acosta and Lepenies, 2019; Alemka et al., 2013; Williams et al., 2020; Moulton et al., 2020). *H. pylori* installs Lewis antigen tetrasaccharides at the terminus of lipopolysaccharide (LPS) in its outer membrane; these epitopes are commonly found on human blood cells and assist the pathogen in evading the host immune response (Wang et al., 2000; Simoons-Smit et al., 1996; Rad et al., 2002). Furthermore, *H. pylori* adhesin protein glycosylation is essential to their function of binding to host gastric epithelial cells (Champasa et al., 2013; Teng et al., 2022). Given previous demonstrations of how bacterial glycans mediate host immune recognition and response, from host recognition of lipopolysaccharide and peptidoglycan by pattern recognition receptors (e.g., Toll-like receptors) to trigger an immune response to bacteria using glycan mimicry to engage immune tolerance receptors (e.g., DC-SIGN) (Alemka et al., 2013; Smedley et al., 2005), we hypothesized that *H. pylori*’s cell surface glycans play a key role in modulating host immune responses by stimulating chronic inflammatory signals and obstructing detection by professional antigen-presenting cells. To assess this hypothesis, we aimed to characterize human immune cell responses to *H. pylori* strains bearing mutations at distinct steps along the glycan biosynthesis pathway.

**Figure 1:**
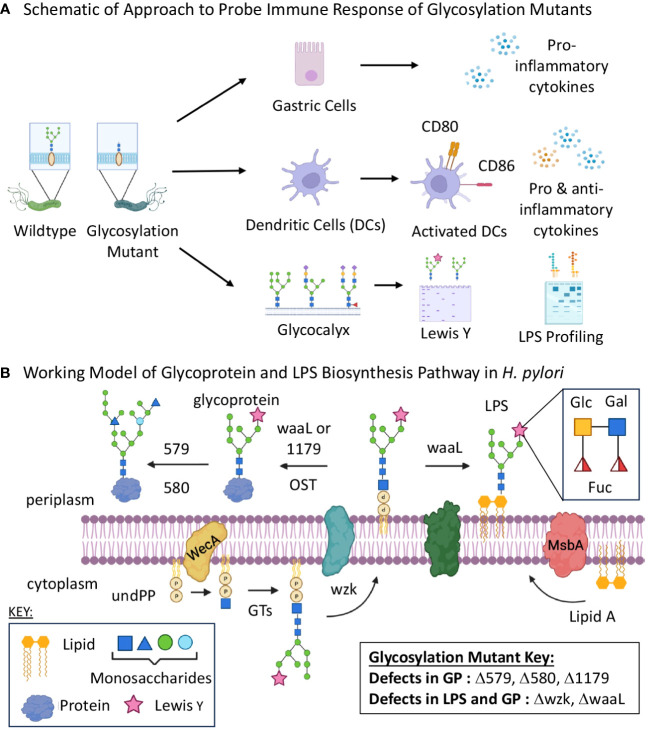
Glycan biosynthesis mutants were screened for immune stimulatory properties. **(A)** Schematic of experimental flow of approach to assessing glycan assembly mutations on host immune recognition and response to *H. pylori*. **(B)** Schematic of putative glycosylation biosynthetic pathway including five genes targeted for mutation and their roles in glycoprotein and lipopolysaccharide assembly. GP, glycoprotein; GT, glycosyltransferase; LPS, lipopolysaccharides; OST, oligosaccharyltransferase; UndPP, undecaprenyl diphosphate. Images were created using BioRender.

Herein we characterize the immune response elicited by five glycosylation mutant strains upon coculture with two models of the human gastric immune microenvironment (**Figure 1A**). We evaluated gastric cytokine production and immune cell maturation when stimulated by each glycosylation mutant relative to wildtype *H. pylori*. Our results demonstrate that strains bearing truncated LPS induced dampened pro-inflammatory cytokine production despite stimulating equivalent levels of immature dendritic cell (iDC) activation compared to wildtype *H. pylori*. Moreover, protein glycosylation mutants induced variable immune responses that did not appear to correlate with phase-variable Lewis Y epitope expression. These results suggest that LPS presentation plays a role in host immune recognition of *H. pylori*, and that the precise role of glycoproteins in driving immune recognition remains unclear. This study demonstrates the need and feasibility for future investigation that probes the relationship between glycocalyx structure and gastric immune response to pathogenic microbes.

## 2 Materials and methods

### 2.1 *H. pylori* strains and bacterial growth.

Wildtype G27 *H. pylori* were grown on horse blood agar plates (HBA) made from 4% Columbia agar base, 5% horse blood, 10 µg/mL of vancomycin, 5 µg/mL cefsulodin, 5 μg/mL trimethoprim, and 8 μg/mL amphotericin B. Bacteria were grown at 37°C in 14% CO_2_. *H. pylori* glycosylation mutants (Δ579, Δ580, Δ1179, Δwzk, ΔwaaL) bearing a chloramphenicol acetyl transferase cassette were grown on HBA plates supplemented with 34 µg/mL of chloramphenicol. Different freezer lots were used in each experiment. Prior to each experiment, wildtype and mutant bacteria were grown for 4-5 days until a full lawn of bacterial growth was observed and diluted to a concentration of 1.0-1.4 x 10 (Chen et al., 2013) cells/mL for cocultures with adenocarcinoma-derived gastric epithelial cells (AGS) or 4.7 x 10 (Kim, 2016) cells/mL for dendritic cell coculture.

### 2.2 Bioinformatics analysis to identify a putative oligosaccharyltransferase gene

With the goal of identifying a putative oligosaccharyltransferase gene involved in *H. pylori’s* general protein glycosylation system, a bioinformatics analysis of *H. pylori* G27 was conducted. Briefly, whole genome alignment was performed using Mauve (version 1.1.3) whole genome alignment on Geneious Prime 2020 (Darling et al., 2004) to identify genes encoding glycosyltransferases that were conserved across other *H. pylori* genomes (G27, 26695, J99, and P12). The G27 genome was analyzed for open reading frames using Glimmer (Delcher et al., 2007) on the KBase Server (Arkin et al., 2018) with the RAST (RASTtk – v1.073) pipeline (Aziz et al., 2008), which was preloaded onto the KBase server with default parameters as “Annotate Microbial Genome.” Genetic analysis of the G27 genomes was performed using Geneious Prime 2020, and the comparison of genes was done using BLAST (Altschul et al., 1990). Domains were identified using HMMER (Eddy, 2011) search against the G27 genomes using the PFam HMM 32.0 database (El-Gebali et al., 2019). For open reading frames that did not have previous biochemical characterization, homology was analyzed using the PHYRE2 recognition server (Kelley et al., 2015). This approach led to the identification of *HpG27_1179* as a putative oligosaccharyltransferase that may play a role in *H. pylori*’s general protein glycosylation system.

### 2.3 Construction of glycosylation mutants and analysis of glycoprotein biosynthesis phenotypes

Strains Δ579 and Δ580 were previously described (Moulton et al., 2020), and Δ1179, Δwzk and ΔwaaL were generated for this study. Gibson assembly was used to generate linear DNA fragments for insertional inactivation of target genes with a chloramphenicol acetyl transferase cassette. The resulting linear DNA was transformed into wildtype strain G27 through natural transformation using the patch method, and gene interruption was autonomously completed by homologous recombination. The successful insertion of the resistance cassette in mutant strains selected on chloramphenicol/HBA agar was confirmed by polymerase chain reaction (PCR) analysis of genomic DNA from selected mutants. A previously reported metabolic glycan labeling strategy using peracetylated *N*-azidoacetylgluocsamine (Ac_4_GlcNAz) and bioorthogonal chemistry with Phos-FLAG was used to detect glycoprotein biosynthesis (Moulton et al., 2020) in wildtype *H. pylori* and newly constructed glycosylation mutants Δ1179, Δwzk and ΔwaaL.

### 2.4 Tissue cell culture

Tissue culture reagents, tissue culture plates, and ELISA kits were purchased from ThermoFisher Scientific (Waltham, MA), MilliporeSigma (Burlington, MA), R&D Systems (Minneapolis, MN), and USA Scientific (Ocala, FL). AGS cells (ATCC Number: CRL-1739) were grown in Ham’s F-12 Glutamax media and passaged upon reaching 80-90% confluency. THP-1 immature dendritic cells (iDCs; ATCC Number: TIB-202) were diluted 1:3 – 1:5 with fresh media (RPMI 1640 with glutamine) when cell concentrations exceeded 1 x 10 (Bravo et al., 2018) cells/mL to prevent overcrowding. Prior to coculture, THP-1 monocytes were differentiated into immature dendritic cells via human recombinant cytokines IL-4 (1500 IU/mL) and GM-CSF (1500 IU/mL) before seeding at a concentration of 2 x 10 (Suerbaum and Michetti, 2002) cells per well in a 6-well plate for coculture. AGS cells were seeded at 5 x 10 (Suerbaum and Michetti, 2002) cells/mL in a 6-well plate before coculture.

### 2.5 Coculture

Wildtype or mutant *H. pylori* were added in 1 mL of tissue culture media to seeded 6-well plates at an MOI of 1:100 for immature dendritic cell challenge or 1:200 for gastric cell culture. Cocultures were incubated for 3-24 hours at 37°C with 5% CO_2_ before harvesting.

### 2.6 ELISA assay

After 3-hour cocultures for AGS cells or 24-hour cocultures for immature dendritic cells, culture medium was collected for analyses. Human Cytokine DuoSet Enzyme Linked Immunoassay (ELISA) kits (R&D Systems) were utilized to detect relative concentrations of CXCL-8 (IL-8), IL-10, TNF- α IL-6, or IL-1 β  conditioned media samples. All cytokine concentration data were determined based on an 8-point standard curve created using recombinant human cytokine standards included in each DuoSet kit.

### 2.7 Flow cytometry analysis of immature dendritic cells

After coculture with wildtype and mutant bacteria, immature dendritic cells were harvested and resuspended in RPMI media with 10% FBS containing FITC-conjugated CD80 and PE-conjugated CD86 goat anti-human antibodies for 30 min on ice (BD Biosciences, Franklin Lakes, NJ). Cells were then washed in PBS and analyzed by flow cytometry using a BD Accuri C6^+^ instrument (BD Biosciences, San Jose, California), with 10,000 live cells gated for each replicate. Dendritic cells expressing CD80 and CD86 were gated and subsequently counted using FlowJo software (TreeStar, Ashland, OR).

### 2.8 Lewis Y Western blot

In parallel to dendritic cell challenge, a same-day plating of the same lot of bacterial cells was used to analyze Lewis Y produced by bacterial cells. Cells were resuspended in *H. pylori* lysis buffer with protease inhibitor [20 mM Tris-HCl, pH 7.4, 1% Igepal, 150 mM NaCl, 1 mM EDTA, Protease inhibitor (MilliporeSigma)] and were frozen for 30 minutes to lyse cells. The protein concentration of lysates was determined using a Lowry assay, and lysate concentrations were standardized to 2.5 mg/ml. Samples were electrophoresed via SDS-PAGE on a Mini-PROTEAN TGX Stain-Free Precast 12% acrylamide gel with 4% stacking layer (Bio-Rad). Coomassie stain was used to evaluate protein loading. For Lewis Y detection, electrophoresed samples were transferred to nitrocellulose for western blot analysis. The nitrocellulose membrane with transferred samples was probed with anti-Lewis Y antibody (Abcam, Waltham, MA) followed by anti-mouse IgM HRP (Southern Biotech, Birmingham, AL), then treated with luminol/peroxidase reagent and visualized using a Syngene G box (Cambridge, UK).

### 2.9 LPS expression profiling

In parallel to dendritic cell challenge, a same-day plating of the same lot of bacterial cells was used to analyze LPS produced by bacterial cells. Bacteria were lysed and diluted in LPS lysis buffer [10% SDS, 4% β-mercaptoethanol, 0.06 mg/mL bromophenol blue, 10% glycerol, 75% 1M Tris-HCl (pH 6.8)] and incubated at 100°C for 10 minutes before cooling to room temperature and treating with Proteinase K (New England Biolabs) at 55°C overnight. To visualize LPS, samples were subsequently electrophoresed alongside a 250 µg/mL *E. coli* LPS (serotype 055:B5) as control via SDS-PAGE using 15% Tris-HCl SDS-PAGE gels. Following electrophoresis, gels were stained using the ProQ Emerald 300 Lipopolysaccharide Gel Stain Kit (Thermo Fisher Scientific) according to manufacturer’s instructions. Gels were visualized with a UVP BioDoc-It Imaging System (Upland, CA) to determine LPS expression at time of coculture.

## 3 Results

### 3.1 LPS elaboration increases pro-inflammatory gastric signaling

An important indicator of immune response and downstream clinical pathology is the secretion of interleukin 8 (IL-8 or CXCL-8) in the gastric microenvironment. CXCL-8 is a pro-inflammatory cytokine that is upregulated to promote an inflammatory response and recruit neutrophils to the site of infection (Harada et al., 1994; Bickel, 1993; Qi et al., 2020). Briefly, gastric epithelial cells express Toll-like receptors (TLRs) on their surfaces that bind to pathogen-associated molecular patterns (PAMP) including bacterial glycans, leading to secretion of CXCL-8 when challenged by bacterial pathogens (Lepper et al., 2005; Smith, 2014; Pachathundikandi et al., 2015; Pachathundikandi and Backert, 2016). Additionally, *H. pylori* can stimulate gastric epithelial cell CXCL-8 production via a Cag type IV secretion system (Cag-T4SS) dependent mechanism, involving ADP-heptose injection into cells, PAMP recognition intracellularly, and NF-kB activation (Faass et al., 2021; Pfannkuch et al., 2019). In *H. pylori* infection, CXCL-8 overexpression is directly linked to gastritis and cancer propagation (Waugh and Wilson, 2008; Liu et al., 2016). Given the importance of CXCL-8 in *H. pylori* pathogenesis, we first sought to determine the effect of modulating *H. pylori*’s glycocalyx on relative CXCL-8 response from gastric cells.

**Figure 2:**
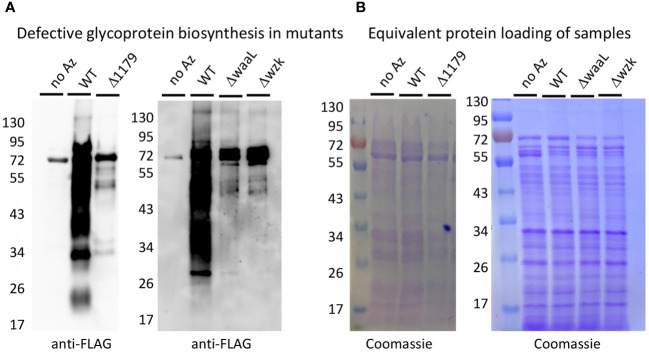
Metabolic labeling revealed that Δ1179, ΔwaaL, and Δwzk exhibit glycoprotein biosynthesis defects. **(A)** Western blot analysis with anti-FLAG antibody revealed robust glycoprotein biosynthesis in wildtype (WT) *H. pylori* treated with Ac_4_GlcNAz (Az) and no apparent azide-dependent signal in the negative control treated with Ac_4_GlcNAc (no Az). Glycosylation mutants Δ*1179*, Δ*waaL*, and Δ*wzk* showed markedly reduced glycoprotein biosynthesis relative to WT when metabolically labeled with Ac_4_GlcNAz. **(B)** Coomassie staining of electrophoresed samples from metabolic glycan labeling experiment revealed that Western samples contained equivalent amounts of protein.

For these experiments, we were particularly interested in the roles of *H. pylori* glycoproteins and LPS in host recognition and response. Thus, we turned to glycosylation genes that play a role in *H. pylori*’s general O-linked protein glycosylation system and/or LPS biosynthesis (**Figure 1B**) (Moulton et al., 2020; Teng et al., 2022; Li et al., 2019; Li et al., 2016). Briefly, previous work in our laboratory and others measured glycoprotein and LPS biosynthesis phenotypes in G27 *H. pylori* mutants bearing insertionally inactivated glycosylation genes (Moulton et al., 2020; Li et al., 2019). These studies identified genes involved in *H. pylori*’s glycoprotein and LPS biosynthesis, as well as provided evidence for initially overlapping glycoprotein and LPS biosynthesis pathways that bifurcate at a later stage (**Figure 1B**). Both pathways appear to begin with glycosyltransferase-catalyzed addition of monosaccharides one-at-a-time onto an undecaprenyl-phosphate lipid-carrier to produce an elaborated lipid-linked glycan on the cytosolic face of the membrane. The elaborated glycan is then flipped across the membrane via the flippase Wzk, then transferred en bloc onto lipid A by the ligase WaaL to produce LPS or onto target proteins (by waaL, or the putative oligosaccharyltransferase (OST) encoded by *HpG27_1179*) to produce glycoproteins. The elaborated glycan on glycoproteins appears to undergo further tailoring by glycosyltransferases encoded by *HpG27_579* and *HpG27_580* (see **Supplementary Table S1** for *H. pylori* 26695 orthologs) (Moulton et al., 2020). Previous studies demonstrated *H. pylori* glycosylation mutants Δ579 and Δ580 are defective in glycoprotein biosynthesis (Moulton et al., 2020) and established that Δwzk and ΔwaaL synthesize core lipid A that lacks O-antigen (Li et al., 2019). To further probe this glycoprotein biosynthesis model, we insertionally inactivated *wzk*, *waaL*, and *HpG27_1179* with the chloramphenicol acetyltransferase cassette and probed glycoprotein biosynthesis in the mutant strains. Using an established metabolic glycan labeling based screen (Moulton et al., 2020), we determined that Δ1179, Δwzk, and ΔwaaL have defects in glycoprotein biosynthesis (**Figure 2**), thus implicating *1179*, *wzk*, and *waaL* in glycoprotein biosynthesis. Armed with this modest panel of five *H. pylori* glycan biosynthesis mutants (Δ579, Δ580, Δwzk, ΔwaaL, Δ1179; **Supplementary Table S1**), we assessed immunogenicity of these strains relative to wildtype bacteria.

**Figure 3:**
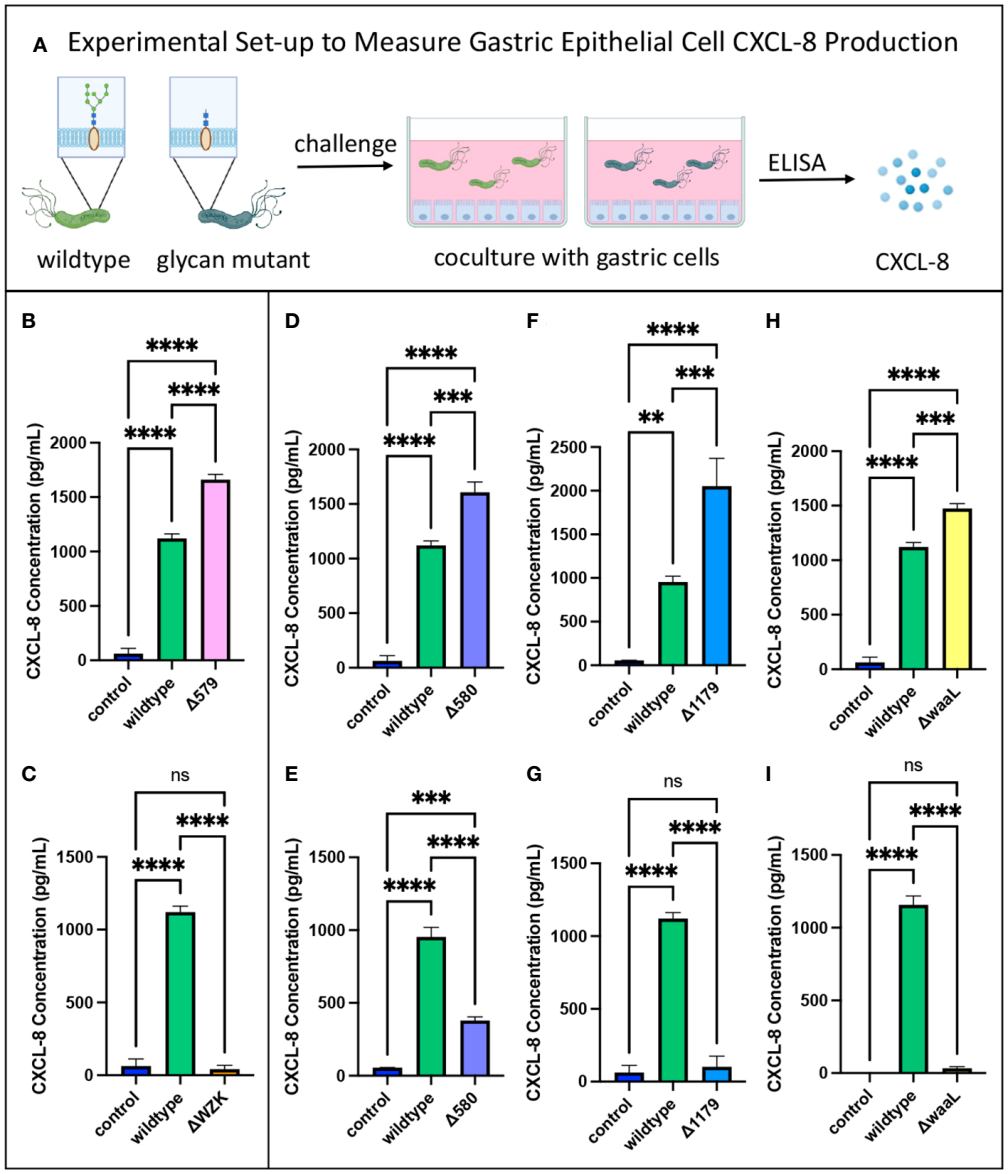
CXCL-8 secretion varied with glycan alterations. **(A)** Experimental workflow for assessing pro-inflammatory cytokine secretion of glycosylation mutants compared to wildtype *H. pylori*. Image was created using BioRender. **(B)** The glycoprotein mutant with elaborated LPS structures, Δ579, induced increased CXCL-8 secretion from gastric epithelial cells relative to wildtype. **(C)** The LPS mutant, Δwzk, induced decreased CXCL-8 secretion from gastric epithelial cells relative to wildtype. **(D–I)** Variable impact on CXCL-8 secretion was observed with glycoprotein mutants Δ580 and Δ1179, and LPS mutant, ΔwaaL, compared to wildtype depending on the coculture experiment. Error bars reflect technical replicates. Tukey’s multiple comparison test one-way ANOVA was used. (**P< 0.01, ***P< 0.001, ****P< 0.0001, ns, not significant). Data are representative of independent replicate experiments (n > 3) that exhibited the same findings.

To assess relative pro-inflammatory response elicited from mutant strains versus wildtype *H. pylori*, gastric epithelial cells were cocultured with the bacterial strains for 3 hours. The supernatants were subsequently collected to measure CXCL-8 secretion levels via an enzyme-linked immunosorbent assay (ELISA) (**Figure 3A**). As a negative control, supernatants were collected from gastric cells cultured in media alone. Supernatants from gastric cells cocultured with wildtype (WT) bacteria acted as a positive control. As expected based on the literature, all wildtype bacteria treatments significantly increased gastric cell secretion of CXCL-8 compared to control cells with no bacterial challenge (**Figure 3**) (El Filaly et al., 2023; Gobert and Wilson, 2022; Outlioua et al., 2020). The glycoprotein mutant with elaborated LPS structures, Δ579, induced consistently increased CXCL-8 secretion from gastric cells compared to wildtype bacteria (**Figure 3B**), whereas the LPS mutant Δwzk led to consistently decreased CXCL-8 secretion (**Figure 3C**). These data are in line with pro-inflammatory properties of fully elaborated LPS engaging TLR4 that may be tempered by interfering with LPS biosynthesis. Diminished inflammatory signaling with LPS biosynthesis interference also aligns with the established mechanism of CXCL-8 induction from Cag T4SS dependent LPS metabolite delivery (Faass et al., 2021; Cover et al., 2020). By contrast, the glycoprotein mutants, Δ580 and Δ1179, and the LPS mutant, ΔwaaL elicited variable CXCL-8 levels that were sometimes significantly higher than wildtype (**Figures 3D, F, H**), and sometimes significantly lower than wildtype and even close to control levels (**Figures 3E, G, I**). Though we were initially surprised by the variability observed in CXCL-8 secretion across biological replicate experiments for Δ580, Δ1179, and ΔwaaL treatments, we conducted a large number of independent experiments to confirm these results. For these mutants, at least three independent replicates led to an increase in CXCL-8 secretion levels relative to wildtype bacteria, and at least three additional biological replicates led to a decrease in cytokine secretion levels relative to wildtype bacteria. The inconsistencies in CXCL-8 response to mutants with similar phenotypic truncations suggests additional factors are at play in modulating CXCL-8 secretion.

To pinpoint immunostimulatory activity of the glycocalyx irrespective of bacterial fitness, we utilized heat-killed bacteria in coculture with gastric epithelial cells. Strikingly, there was no upregulation of CXCL-8 secretion from gastric cells challenged with heat-killed *H. pylori* (**Supplementary Figure S1**). Instead, negative control levels of CXCL-8, on par with the addition of no bacteria, were released by gastric cells treated with heat-killed wildtype *H. pylori* or heat-killed Δ580 cells (**Supplementary Figure S1**). These results indicate that active infection of bacteria and the presentation of an integrated glycocalyx may be essential to immune-stimulatory responses.

### 3.2 Dendritic cells activate against *H. pylori* glycosylation mutants

Dendritic cells play a central role in the formation of a cell-mediated, adaptive immune response. These cells balance immune tolerance through binding regulatory T cells with immune reaction through phagocytosis and antigen-presentation to CD4 and CD8 T cells (Coombes and Powrie, 2008). *H. pylori* induces strong activation and maturation of human dendritic cells at levels comparable to the LPS of *E. coli* (Kranzer et al., 2004). However, it is not clear how *H. pylori* modulates regulation of dendritic cell maturation, nor how this modulation may affect the role of dendritic cells in discerning pathogens from commensal gut microbes. Some studies suggest that relatively low immunostimulatory activity of *H. pylori* LPS contributes to *H. pylori*’s apparent immune evasion (Pérez-Pérez et al., 1995), while other studies suggest a shift in downstream pathways promotes regulatory T cell binding and immune tolerance to the pathogen (Kao et al., 2010).

In either case, we aimed to complement our innate immune response findings from AGS cells with a more direct measure of adaptive immune modulation via dendritic cell maturation. As professional antigen-presenting cells, dendritic cells traverse the gastric epithelial layer, surveying the gastrointestinal tract until met with a pathogen. Upon contact and recognition, immature dendritic cells are activated, altering their morphology to activate both CD4 and CD8 T cells for a robust adaptive immune response (Liu and Cao, 2015). As part of this transformation, they upregulate cell-surface coregulatory receptors CD80 and CD86, which bind CD28 on CD4^+^ and CD8^+^ T cells. These biomarkers may be detected as a proxy for immune cell activation, as they are expressed at low levels on immature dendritic cells (Kim and Kim, 2019). The percentage of iDC activation reflects the extent of adaptive immune response. High CD80 and CD86 expression levels generated by wildtype *H. pylori* correspond to higher recognition and increased downstream adaptive immune responses, including cytotoxic T cell development.

**Figure 4:**
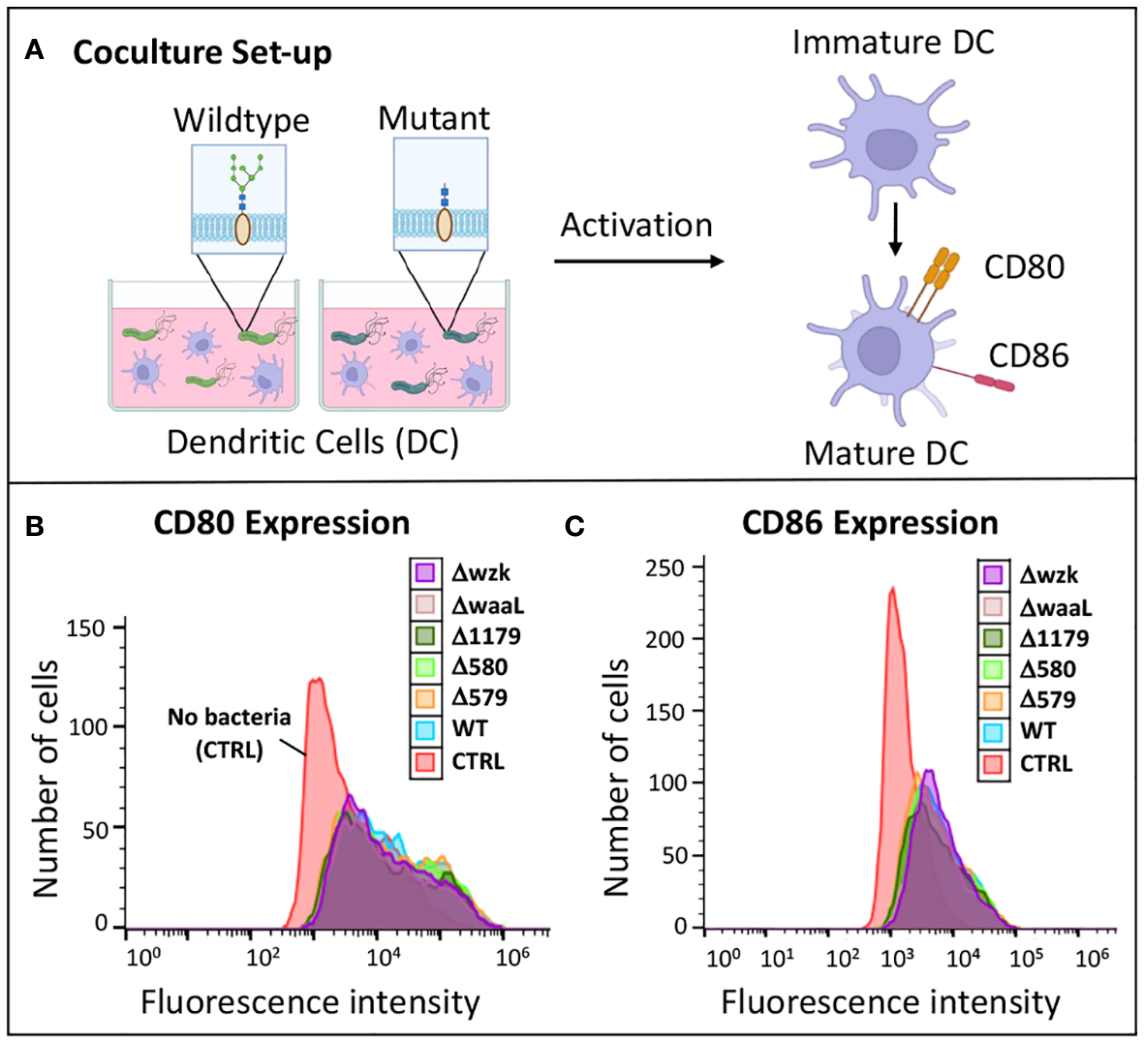
Immature dendritic cells became activated when challenged with wildtype *H. pylori* and glycosylation mutants. **(A)** Experimental workflow for assessing iDC activation upon challenge with glycosylation mutant bacteria compared to wildtype. Image was created using BioRender. **(B, C)** Flow cytometry histograms revealed CD80 and CD86 expression of iDCs was increased upon challenge of cells with glycosylation mutants and wildtype *H. pylori* relative to iDCs treated with no bacterial (CTRL). Data are representative of independent replicate experiments (n > 3).

To test for an effect of glycan truncation on immune cell activation, immature dendritic cells were challenged with wildtype *H. pylori* and glycosylation mutants, then their relative immune response was determined via flow cytometry analysis. After 24 hours in coculture with bacteria, iDCs were isolated, probed with anti-CD80 and anti-CD86 antibodies, and analyzed by flow cytometry to measure fluorescence (**Figure 4A**). Higher fluorescence indicates higher biomarker expression levels, corresponding to an increased immune response by iDCs. iDCs cultured with no bacteria served as a negative control. Flow cytometry analysis revealed that dendritic cells challenged with bacteria exhibited significantly increased CD80 and CD86 biomarker expression relative to unchallenged dendritic cells (**Figure 4**; **Supplementary Figure S2**). Flow cytometry histograms of iDCs challenged with wildtype *H. pylori* overlaid closely with iDCs challenged with glycosylation mutants (**Figure 4**). There were no significant differences in CD80 and CD86 levels on iDCs, or with percent of cells with high expression levels, following challenge with wildtype *H. pylori* versus glycosylation mutants (**Supplementary Figure S2**). These findings indicate an upregulation of cell-mediated immune response to *H. pylori* glycosylation mutants tested that was on par with activation stimulated by wildtype *H. pylori* (**Figures 4B, C**; **Supplementary Figure S2**).

**Figure 5:**
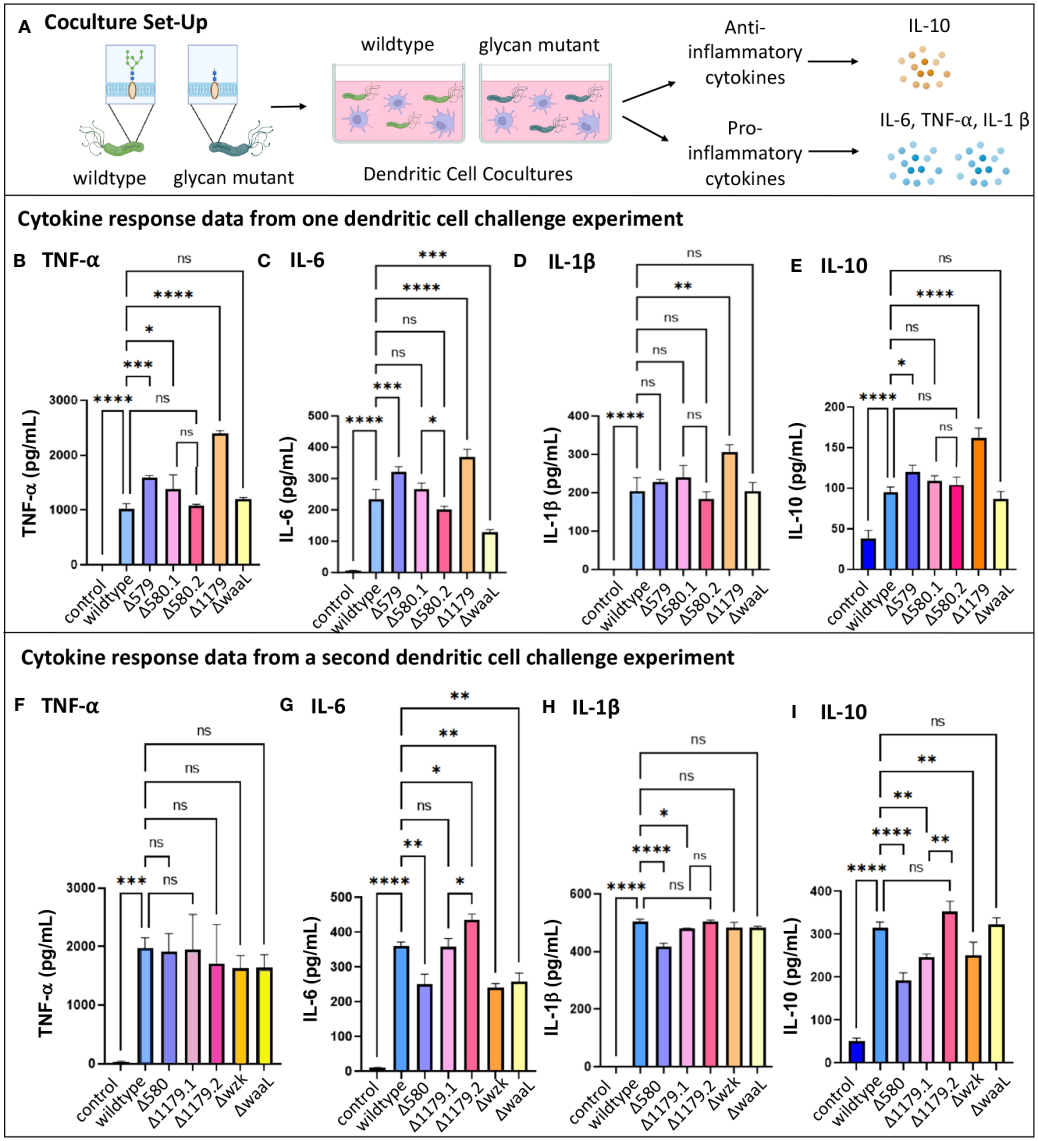
Immature dendritic cell cytokine secretion changed when challenged by *H. pylori* glycosylation mutants. **(A)** Experimental workflow used to assess TNF-α, IL-6, IL-1β, and IL-10 secretion from iDCs following challenge for 24-hours with glycosylation mutants or wildtype bacteria. Image was created using BioRender. Datasets from two different iDC challenge experiments are shown, with **(B–E)** collected in one experiment and **(F–I)** collected in a second experiment. Δ580.1 and Δ 580.2, as well as Δ1179.1 and Δ1179.2, represent different freeze lots of the same strain. Error bars reflect technical replicates. Tukey’s multiple comparison test one-way ANOVA was used. (*P< 0.05, **P< 0.01, ***P< 0.001, ****P< 0.0001, ns, not significant).

We next measured levels of cytokine secretion by dendritic cells in response to 24-hour challenge with wildtype *H. pylori* versus glycosylation mutants (**Figure 5A**). Though iDCs exposed to wildtype and mutant *H. pylori* strains became activated to a similar extent, they secreted markedly different cytokine levels in response to wildtype versus glycosylation mutants (**Figure 5**). Secretion of the pro-inflammatory cytokines TNF- α, IL-1β and IL-6, as well as the anti-inflammatory cytokine IL-10, by iDCs revealed similar relative patterns of cytokine secretion levels in response to challenge across mutant samples in a single experiment (**Figures 5B–E, G, I**). In all experiments, the LPS mutants ΔwaaL and Δwzk both induced lower IL-6 secretion from dendritic cells relative to wildtype challenge (**Figures 5C, G**), with levels secreted by ΔwaaL on par to wildtype-induced secretion for TNF- α (**Figures 5B, F**) and IL-1β (**Figures 5D, H**). In contrast, the glycoprotein mutant Δ579 significantly increased TNF- α and IL-6 secretion compared to wildtype coculture (**Figures 5B, C**). The trends suggest diminished secretion of pro-inflammatory cytokines in the absence of LPS, which mirror the findings from gastric epithelial pro-inflammatory cytokine modulation. In contrast, anti-inflammatory cytokine IL-10 secretion levels were not altered in a consistent manner upon treatment with glycosylation mutants relative to wildtype challenge (**Figures 5E, I**).

Taken together, the results of these studies indicate that dendritic cells are activated by *H. pylori* glycosylation mutants, and the extent of cytokine secretion varies based on the glycocalyx expression profile. As with the gastric cell results, pro-inflammatory cytokine responses are somewhat diminished in coculture with bacteria bearing truncated LPS (ΔwaaL and Δwzk) and enhanced in coculture with mutants displaying fully elaborated LPS but reduced glycoprotein (Δ579). Notably, all mutant *H. pylori* bearing an impaired glycocalyx induce immature dendritic cell immune recognition and activation on par with the wildtype pathogen. Alterations in iDC cytokine secretion, in particular levels of IL-6 and IL-10, induced by glycosylation mutants suggest that glycan structure may modulate downstream polarization and the downstream CD4 response.

As with variable CXCL-8 secretion observed in challenge experiments with AGS cells (**Figures 3D–I**), we observed high variability in relative levels of dendritic cell cytokine secretion across biological replicates (**Figure 5**). Challenge of iDCs with different lots of Δ1179 (Δ1179.1 and Δ1179.2) in the same experiment led to large differences in IL-6 and IL-10 secretion for these lots relative to one another (**Figures 5G, I**) and across experiments relative to wildtype bacteria (**Figure 5**). More subtly, challenge with different lots of Δ580 (Δ580.1 and Δ580.2) in the same experiment led to significantly different IL-6 secretion from iDCs for those lots relative to one another (**Figure 5C**). Further, Δ580 elicited variable cytokine secretion across experiments, with no clear trends in cytokine secretion relative to wildtype bacterial challenge (**Figure 5**). Variation across replicates in both the gastric cell and dendritic cell models could be due to phase variation in expression of glycan epitopes, heterogeneity in the mutant population, variations in virulence factors such as Cag T4SS activity, or technical problems. Given the well-established literature of phase variable glycocalyx construction in *H. pylori*, we reasoned that further assessment of mutant glycocalyx expression at the time of coculture was warranted.

### 3.3 Immune response correlates with extent of LPS O-antigen elaboration

The variable cytokine secretion induced by glycosylation mutants in both the gastric and dendritic cell cocultures (**Figures 3D–I, 5**) prompted us to probe whether phase variable expression of glycan epitopes might be eliciting inconsistent immune responses across experiments. Phase variation is a characteristic of many prokaryotic microbes in which they employ selective gene expression for proteins from generation to generation. In essence, phase variation is a reversible switch between an “on” or “off” expression phase, which manipulates the level of expression for one or more proteins in microbes of a clonal population (van der Woude and Bäumler, 2004). Phase variation is well established in *H. pylori* and related pathogens. Expression of Lewis blood-group antigens on LPS may vary within a single strain of *H. pylori* as a result of high-frequency on/off switching of fucosyltransferase genes involved in LPS biosynthesis (Wang et al., 2000; van der Woude and Bäumler, 2004; Bergman et al., 2006). We hypothesized that the variable pro-inflammatory cytokine secretion of host cells in response to challenge by Δ580, Δ1179, and ΔwaaL could reflect the structure of the glycocalyx at the time of challenge.

**Figure 6:**
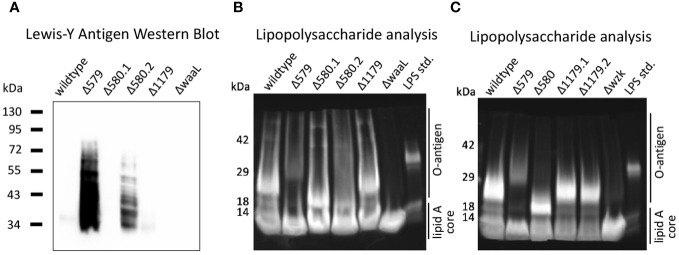
The glycocalyx was probed to assess how Lewis Y expression and LPS elaboration correlate to cytokine secretion by immune cells. **(A)** Western blot analysis reveals robust Lewis Y expression in Δ579 and Δ580.2. The strain samples used in these experiments correspond to those used in iDC challenge experiments for the dataset shown in **Figure 5**. **(B, C)** Analysis of LPS by gel electrophoresis reveals that Δwzk and ΔwaaL synthesize only truncated, low molecular weight LPS relative to wildtype *H. pylori*, whereas Δ579 appears to synthesize LPS with somewhat more prominent bands at higher molecular weights (e.g., >29kDa) than wildtype *H. pylori*. **(B)** The strain samples used in these experiments correspond to those used in dendritic cell challenge experiments for the dataset shown in **Figure 5**. Δ580.1 and Δ 580.2 represent different freeze lots of the same strain. **(C)** Analysis of LPS by gel electrophoresis for strain samples used in dendritic cell challenge experiments for the dataset shown in **Supplementary Figure S3**. Δ1179.1 and Δ1179.2 represent different freeze lots of the same strain and appear to have comparable LPS fingerprints.

Lewis Y is an established phase-variable epitope whose level of expression is linked to a strand slippage event that controls fucosyltransferase activity in *H. pylori*. Curious about the expression of the Lewis Y tetrasaccharide in glycosylation mutants used in immune challenge experiments, we determined relative expression of the Lewis Y antigen of mutants used in dendritic cell challenge experiments (**Figure 5**). Western blot analysis revealed Lewis Y expression varied dramatically across *H. pylori* strains despite equivalent protein levels (**Supplementary Figure S3A**). Two strains, Δ579 and Δ580.2, exhibited robust Lewis Y expression (**Figure 6A**). Conversely, Δ580.1, Δ1179, ΔwaaL, and wildtype cells displayed minimal Lewis Y expression (**Figure 6A**). These results are consistent with variable Lewis Y expression on LPS structures of wildtype *H. pylori* (Simoons-Smit et al., 1996; Bergman et al., 2006).

Notably, two freeze lots of the Δ580 glycoprotein mutant, Δ580.1 and Δ580.2, demonstrated distinctly different expression levels of Lewis Y (**Figure 6A**). Such variation between lots of the same glycan mutant is consistent with previous evidence that the *H. pylori* glycocalyx displays antigenic variation within a single strain or clinical isolate (Wirth et al., 1999). For example, wildtype *H. pylori*, even the same strain, exhibits phase-variable Lewis Y expression (Wirth et al., 1999). Thus, the difference in Lewis Y expression in Δ580.1 and Δ580.2 is likely due to phase variation. Further, these data are concordant with the possibility of phase variable epitopes influencing immune recognition and response. However, when we scrutinized the correlation between Lewis Y expression and relative cytokine secretion from the same samples in our dendritic cell model systems, we observed no clear trend. In particular, Δ579 and Δ1179 exhibited disparate Lewis Y expression (**Figure 6A**) yet both induced heightened levels of pro-inflammatory cytokine secretion in immature dendritic cell challenge experiments (**Figure 5**). Similarly, drastically different Lewis Y expression from Δ580.1 and Δ580.2 (**Figure 6A**) yielded similar IL-1β and IL-10 secretion levels relative to each other and wildtype bacteria (**Figure 5**). These results suggest that Lewis Y expression is not a principal factor driving pro-inflammatory cytokine secretion from dendritic cells. It is likely that some other epitope is more important.

LPS structures similarly undergo phase variation in *H. pylori*’s glycocalyx, where the expression of the glycosyltransferase enzymes responsible for O-antigen elaboration may be turned on or off (Appelmelk and Vandenbrouck-Grauls, 2003; Lukáčová et al., 2008). Understanding that LPS is a mediator of *H. pylori’*s interactions in its environment, we sought to investigate the extent of elaboration of LPS produced by mutant and wildtype bacteria at the time of immune challenge as an additional parameter to contextualize immune response with glycan architecture. We hypothesized that the extent of LPS elaboration at the time of coculture would correlate with level of immune response. Crude LPS was isolated from bacterial samples used in the dendritic cell experiment in **Figures 5B–E** and **5F–I**, and compared to literature fingerprints for *H. pylori* LPS (Li and Benghezal, 2017). Briefly, bacterial lysates were analyzed by Coomassie stain to confirm equivalent protein loading (**Supplementary Figure S3**), then treated with proteinase K to yield crude LPS. LPS samples were electrophoresed and their molecular weight distribution was visualized alongside an *Escherichia coli* LPS standard (**Figures 6B, C**). The Δ579 LPS fingerprint exhibited bands at higher molecular weights (e.g., >29 kDa) than wildtype *H. pylori*, implying this mutant synthesized more elaborated O-antigen. This strain also induced higher CXCL-8 levels (**Figure 3**), possibly implicating elaborated O-antigen presentation in higher CXCL-8 levels. We noted that bands corresponding to high molecular weight O-antigen in Δ579 were less prominent than lower molecular weight O-antigen produced by wildtype *H. pylori*, suggesting there may be a heterogeneous population of Δ579 with different phase types. In contrast to elaborated LPS produced by Δ579, LPS produced by ΔwaaL and Δwzk was limited to a prominent band at<14 kDa corresponding to the lipid A core, thus confirming truncated LPS biosynthesis (**Figures 6B, C**). However, the glycoprotein mutant Δ580 had variable LPS elaboration, with differences in relative production of high molecular weight O-antigen (**Figures 6B, C**).

Within the context of immune response data, we noticed apparent correlations with extent of LPS elaboration and extent of cytokine secretion elicited. For example, Δ579 displayed relatively high molecular weight LPS structures (**Figures 6B, C**) and elicited heightened pro- and anti-inflammatory cytokine secretion from challenged host cells (**Figures 3B**, **5B, C, E**). By contrast, Δwzk and ΔwaaL displayed truncated LPS (**Figures 6B, C**) and elicited somewhat mitigated IL-6 secretion from challenged host cells (**Figure 5**). Taken together, these data indicate that relative elaboration of LPS may play a role in extent of immune recognition and response by dendritic cells, but more detailed methods need to be developed for LPS analysis to fully support this claim. The role of *H. pylori’*s general protein glycosylation system in modulating immune response is less clear.

## 4 Discussion

**Figure 7:**
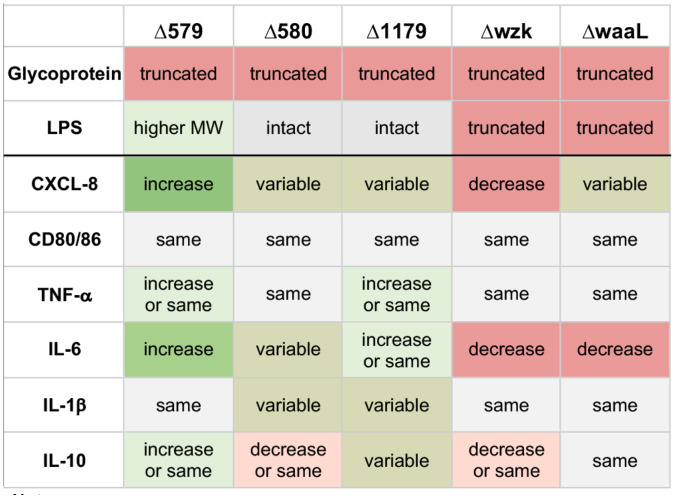
Summary of immune responses elicited by challenge with glycosylation mutants of *H. pylori* bearing defects in glycoprotein and/or LPS biosynthesis, as indicated. Effects are relative to immune response elicited by challenge with wildtype *H. pylori* in the same experiment. Data are based on biological replicates (n > 3). “Truncated” glycoprotein indicates reduction in glycoprotein biosynthesis relative to wildtype; “Truncated” LPS indicates lower molecular weight LPS biosynthesized than wildtype; “Higher MW” LPS indicates higher molecular weight LPS biosynthesized than wildtype; “Intact” LPS indicates LPS with similar molecular weight pattern as wildtype; “Increase” indicates a significant increase relative to wildtype; “Decrease” indicates a significant decrease relative to wildtype; “Same” reflects equivalent levels relative to wildtype; “Variable” indicates the effect depended on the replicate, with significant increases and decreases observed.

*H. pylori* are a compelling pathogen to study in the context of the immune system. Previous work showed that *H. pylori* incorporate Lewis antigen epitopes within the terminus of their LPS structures, suggesting that their glycans feign self in the host environment (Wang et al., 2000; Simoons-Smit et al., 1996; Lee et al., 2006). Given the established role that LPS plays in immune stimulation by similar pathogens, and the characterized role of *H. pylori*’s glycans in bacterial fitness and binding to host cells, our study aimed to investigate the role of *H. pylori* cell surface glycosylation on host immune cell signaling and response. Here, we surveyed a small panel of glycosylation mutants bearing insertional inactivation of genes implicated in glycan biosynthesis (Moulton et al., 2020) for their ability to invoke an immune response (**Figure 7**). Our data implicate extent of *H. pylori* LPS elaboration with extent of host immune response, with glycoprotein elaboration playing a less clear role. This work offers a novel approach to assess the role of *H. pylori* glycan biosynthesis in modulating host immune recognition and response.

Gastric epithelial cells produced heightened levels of the pro-inflammatory cytokine CXCL-8 upon challenge with Δ579, a glycoprotein mutant that synthesizes elaborated LPS (**Figure 3B**). In contrast, Δwzk, which bears truncated LPS and glycoproteins, induced decreased CXCL-8 expression relative to wildtype bacteria. These data support previous findings that attribute epithelial cell cytokine production to bacterial LPS stimulation (Lotz et al., 2007). It is well established that CXCL-8 is produced by gastric cells during *H. pylori* infection (Eftang et al., 2012). In addition, CXCL-8 is linked to gastric cancer proliferation; it promotes chronic inflammation and propagates infection (Waugh and Wilson, 2008; Liu et al., 2016). In fact, CXCL-8 levels positively correlate to poor clinical outcomes for patients who have gastric cancer, decreasing CD8+ T cell infiltration and increasing immunosuppressor programmed death ligand 1 (PD-L1) expression on macrophages (Lin et al., 2019). Simultaneously, LPS has been found to stimulate significant upregulation of the cannabinoid receptor 1 in the gastrointestinal tract, which promotes cancer cell proliferation (Sedighzadeh et al., 2020). Other studies implicate LPS in cancer immune suppression, demonstrating that LPS contributes to T cell exhaustion and upregulates PD-L1 in lung cancer, establishing a need to explore this link in gastric cancer (Liu et al., 2021; Shi et al., 2022). Our data add nuance to these reports by implicating *H. pylori* LPS with CXCL-8 upregulation, suggesting possible roles for glycan structures in pathogenesis, chronic infection, and cancer development upon infection.

Our study demonstrates that alteration and truncation of glycan structures on *H. pylori*’s cell surface do not hinder the activation of immature dendritic cells and their concomitant expression of CD80 and CD86 (**Figure 4**), consistent with previous reports measuring iDC activation via biomarker upregulation (Kranzer et al., 2004). We found that glycan alteration and truncation yielded differences in cytokine expression profiles from iDCs (**Figure 5**). These results indicate that the downstream activation and polarization of CD4 T cells might be different between the different glycosylation mutants. As such, there may be value in assessing glycan structures, the type of adaptive immune response triggered, and the extent to which downstream responses are useful against extracellular pathogens or harmful to the host. Follow up studies could reveal glycan targets that modulate inflammatory cytokine signaling and downstream immune response while maintaining upregulated CD80/CD86 expression in ways that benefit the host.

A confounding observation in our studies was the variable cytokine signaling responses across multiple freeze lots of the same glycosylation mutant or of the same mutant in biological replicates (e.g., Δ580, Δ1179 and ΔwaaL; **Figures 3**, **5**). These results prompted us to probe phase variable Lewis Y presentation in our *H. pylori* mutants to assess whether there is a correlation between Lewis Y expression and relative immune response. Previous reports implicate the tetrasaccharide blood-group antigen Lewis Y in *H. pylori* immune modulation, evasion, and pathogenicity (Mandrell and Apicella, 1993; Appelmelk et al., 2000; Bergman et al., 2004; van der Woude and Bäumler, 2004). Some attribute *H. pylori* colonization persistence and homeostasis between host and pathogen to the phase variable expression of Lewis blood-group antigens, suggesting a commensal origin commandeered for opportunistic pathogenicity (Bergman et al., 2006). To query the effect of Lewis Y variation on our mutant immune response data, we probed Lewis Y expression in bacterial lysates used in iDC challenge experiments. We observed no clear correlation with Lewis Y expression and cytokine secretion (**Figures 5**, **6**). This result was surprising due to the reported role of Lewis Y in engaging DC-SIGN on dendritic cells to suppress immune response (Bergman et al., 2004). Our data suggest that Lewis Y does not appear to be the sole proprietor for regulating *H. pylori-*induced pro-inflammatory responses and suggest that other genes are regulated by the phase variation phenomena.

There appears to be a more direct connection between LPS elaboration and immunogenicity. Although previous studies have demonstrated that *H. pylori* LPS is less endotoxic compared to other bacterial counterparts, the pathogen’s modified lipid A and O-antigen are believed to permit selectivity of binding to certain host receptor molecules, enabling the bacterium’s persistence (Cullen et al., 2011; Chmiela et al., 2014; Lina et al., 2014). Our results provide novel insight into the role of *H. pylori* LPS in the context of whole cells. In particular, elaborated LPS present on Δ579 appears to be correlated with increased pro-inflammatory cytokines including CXCL-8 (**Figure 3**), TNF-α, and IL-6 (**Figure 5**). Furthermore, truncation of the LPS moiety correlates to somewhat mitigated cytokine secretion (e.g., IL-6) from challenged host cells (**Figures 3**, **5**).

The precise mechanisms by which *H. pylori* bearing elaborated LPS and glycoproteins engage immune receptors was not explored in this work. Expanding our understanding of the structures of *H. pylori*’s glycans and their influence on immune recognition and response will allow us to pinpoint structures, and their corresponding biosynthesis enzymes, that contribute to the pathogen’s engagement of immune receptors. Further, determining the relative heterogeneity of glycans in a single *H. pylori* population, assessing the phenotypes of complemented strains, and probing immune response elicited by independent mutants are important future directions. Moreover, exploring the total number of protein antigens modified by glycans in wildtype bacteria versus glycosylation mutants, and probing how glycan modification events impact antigenicity of proteins, relative protein stability, and protein abundance, will be important steps to tease out the molecular mechanisms by which *H. pylori*’s general protein glycosylation system influences host immune responses. Our results suggest the potential to manipulate host immune tolerance and activation through targeting select glycans on *H. pylori*’s cell surface to quell pro-inflammatory responses characteristic of gastritis and gastric cancer while maintaining dendritic cell activation necessary for adaptive immune system upregulation. Finally, our results indicate that *H. pylori* LPS plays an important role in stimulating the immune response.

## 5 Conclusion

*H. pylori* is a gut pathogen that evades clearance by the immune system. *H. pylori’s* glycans play an essential role in the bacteria’s ability to colonize and thrive in the gastrointestinal tract (Rad et al., 2002; Schirm et al., 2003; Lina et al., 2014; Moulton et al., 2020). This work investigates the role of cell surface glycans in modulating host immune response to *H. pylori* and demonstrates the potential for selective perturbation of glycan structures to diminish pro-inflammatory responses in severe infections that lead to gastritis and gastric cancer. These studies offer insight into immune response data in conjunction with *H. pylori* glycan architecture and antigen presentation. Broadly, this work suggests that glycan-specific targeting of *H. pylori* could pose a means to dampen chronic inflammatory responses while maintaining cell-mediated adaptive immune system upregulation.

## Data availability statement

The raw data supporting the conclusions of this article will be made available by the authors, without undue reservation.

## Ethics statement

Ethical approval was not required for the studies on humans in accordance with the local legislation and institutional requirements because only commercially available established cell lines were used.

## Author contributions

KB: Formal analysis, Investigation, Methodology, Validation, Visualization, Writing – original draft, Writing – review & editing. FK: Conceptualization, Formal analysis, Investigation, Methodology, Validation, Writing – review & editing. WS: Formal analysis, Investigation, Methodology, Validation, Visualization, Writing – review & editing. AM: Investigation, Methodology, Writing – review & editing. KM: Investigation, Methodology, Project administration, Supervision, Writing – review & editing. DD: Conceptualization, Funding acquisition, Project administration, Supervision, Writing – original draft, Writing – review & editing.

## References

[B1] Alemka A. Nothaft H. Zheng J. Szymanski C. M. (2013). N-glycosylation of *campylobacter jejuni* surface proteins promotes bacterial fitness. Infect. Immun. 81, 1674–1682. doi: 10.1128/IAI.01370-12 23460522 PMC3648013

[B2] Altschul S. F. Gish W. Miller W. Myers E. W. Lipman D. J. (1990). Basic local alignment search tool. J. Mol. Biol. 215, 403–410. doi: 10.1016/S0022-2836(05)80360-2 2231712

[B3] Appelmelk B. J. Martino M. C. Veenhof E. Monteiro M. A. Maaskant J. J. Negrini R.. (2000). Phase variation in H type I and lewis a epitopes of *helicobacter pylori* lipopolysaccharide. Infect. Immun. 68, 5928–5932. doi: 10.1128/IAI.68.10.5928-5932.2000 10992504 PMC101556

[B4] Appelmelk B. J. Vandenbrouck-Grauls C. M. J. E. (2003). “7 - phase variation in *helicobacter pylori* lipopolysaccharide,” in Antigenic variation. Eds. Craig A. Scherf A. (Academic Press, London), 122–141.

[B5] Arkin A. P. Cottingham R. W. Henry C. S. Harris N. L. Stevens R. L. Maslov S.. (2018). KBase: the United States department of energy systems biology knowledgebase. Nat. Biotechnol. 36, 566–569. doi: 10.1038/nbt.4163 29979655 PMC6870991

[B6] Aziz R. K. Bartels D. Best A. A. DeJongh M. Disz T. Edwards R. A.. (2008). The RAST server: rapid annotations using subsystems technology. BMC Genomics 9, 75. doi: 10.1186/1471-2164-9-75 18261238 PMC2265698

[B7] Baj J. Forma A. Sitarz M. Portincasa P. Garruti G. Krasowska D.. (2020). *Helicobacter pylori* virulence factors—Mechanisms of bacterial pathogenicity in the gastric microenvironment. Cells 10, 27. doi: 10.3390/cells10010027 33375694 PMC7824444

[B8] Bergman M. Del Prete G. van Kooyk Y. Appelmelk B. (2006). *Helicobacter pylori* phase variation, immune modulation and gastric autoimmunity. Nat. Rev. Microbiol. 4, 151–159. doi: 10.1038/nrmicro1344 16415930

[B9] Bergman M. P. Engering A. Smits H. H. van Vliet S. J. van Bodegraven A. A. Wirth H.-P.. (2004). *Helicobacter pylori* modulates the T helper cell 1/T helper cell 2 balance through phase-variable interaction between lipopolysaccharide and DC-SIGN. J. Exp. Med. 200, 979–990. doi: 10.1084/jem.20041061 15492123 PMC2211851

[B10] Bickel M. (1993). The role of interleukin-8 in inflammation and mechanisms of regulation. J. Periodontol 64, 456–460. 8315568

[B11] Bravo D. Hoare A. Soto C. Valenzuela M. A. (2018). Quest, A. F. *Helicobacter pylori* in human health and disease: mechanisms for local gastric and systemic effects. World J. Gastroenterol. 24, 3071–3089. doi: 10.3748/wjg.v24.i28.3071 30065554 PMC6064966

[B12] Byrd J. C. Yunker C. K. Xu Q. Sternberg L. R. Bresalier R. S. (2000). Inhibition of gastric mucin synthesis by *helicobacter pylori* . Gastroenterology 118, 1072–1079. doi: 10.1016/S0016-5085(00)70360-X 10833482

[B13] Cellini L. Grande R. Di Campli E. Traini T. Di Giulio M. Nicola Lannutti S.. (2008). Dynamic colonization of *helicobacter pylori* in human gastric mucosa. Scandinavian J. Gastroenterol. 43, 178–185. doi: 10.1080/00365520701675965 17918004

[B14] Champasa K. Longwell S. A. Eldridge A. M. Stemmler E. A. Dube D. H. (2013). Targeted identification of glycosylated proteins in the gastric pathogen *helicobacter pylori* (*Hp*). Mol. Cell Proteomics 12, 2568–2586. doi: 10.1074/mcp.M113.029561 23754784 PMC3769331

[B15] Chen Y. Segers S. Blaser M. J. (2013). Association between *helicobacter pylori* and mortality in the NHANES III study. Gut 62, 399–403. doi: 10.1136/gutjnl-2012-303018 PMC3834579 23303440

[B16] Chmiela M. Miszczyk E. Rudnicka K. (2014). Structural modifications of *helicobacter pylori* lipopolysaccharide: an idea for how to live in peace. World J. Gastroenterol. 20, 9882–9897. doi: 10.3748/wjg.v20.i29.9882 25110419 PMC4123370

[B17] Coombes J. L. Powrie F. (2008). Dendritic cells in intestinal immune regulation. Nat. Rev. Immunol. 8, 435–446. doi: 10.1038/nri2335 18500229 PMC2674208

[B18] Cover T. L. Blanke S. R. (2005). *Helicobacter pylori* vacA, a paradigm for toxin multifunctionality. Nat. Rev. Microbiol. 3, 320–332. doi: 10.1038/nrmicro1095 15759043

[B19] Cover T. L. Lacy D. B. Ohi M. D. (2020). The *helicobacter pylori* cag type IV secretion system. Trends Microbiol. 28, 682–695. doi: 10.1016/j.tim.2020.02.004 32451226 PMC7363556

[B20] Cullen T. W. Giles D. K. Wolf L. N. Ecobichon C. Boneca I. G. Trent M. S. (2011). *Helicobacter pylori* versus the host: remodeling of the bacterial outer membrane is required for survival in the gastric mucosa. PloS Pathog. 7, e1002454. doi: 10.1371/journal.ppat.1002454 22216004 PMC3245313

[B21] Darling A. C. E. Mau B. Blattner F. R. Perna N. T. (2004). Mauve: multiple alignment of conserved genomic sequence with rearrangements. Genome Res. 14, 1394–1403. doi: 10.1101/gr.2289704 15231754 PMC442156

[B22] Delcher A. L. Bratke K. A. Powers E. C. Salzberg S. L. (2007). Identifying bacterial genes and endosymbiont DNA with glimmer. Bioinformatics 23, 673–679. doi: 10.1093/bioinformatics/btm009 17237039 PMC2387122

[B23] Di Fermo P. Di Lodovico S. Di Campli E. D’Arcangelo S. Diban F. D’Ercole S.. (2023). *Helicobacter pylori* dormant states are affected by vitamin C. Int. J. Mol. Sci. 24, 5776. doi: 10.3390/ijms24065776 36982855 PMC10057322

[B24] Dong B. N. Graham D. Y. (2017). *Helicobacter pylori* infection and antibiotic resistance: A WHO high priority? Nat. Rev. Gastroenterol. Hepatol. 14, 383–384. doi: 10.1038/nrgastro.2017.57 28465548 PMC6905073

[B25] Doohan D. Rezkitha Y. A. A. Waskito L. A. Yamaoka Y. (2021). Miftahussurur, M. *Helicobacter pylori* babA–sabA key roles in the adherence phase: the synergic mechanism for successful colonization and disease development. Toxins 13, 485. doi: 10.3390/toxins13070485 34357957 PMC8310295

[B26] Dunne C. Dolan B. Clyne M. (2014). Factors that mediate colonization of the human stomach by *helicobacter pylori* . World J. Gastroenterol. 20, 5610–5624. doi: 10.3748/wjg.v20.i19.5610 24914320 PMC4024769

[B27] Eddy S. R. (2011). Accelerated profile HMM searches. PloS Comput. Biol. 7, e1002195. doi: 10.1371/journal.pcbi.1002195 22039361 PMC3197634

[B28] Eftang L. L. Esbensen Y. Tannæs T. M. Bukholm I. R. Bukholm G. (2012). Interleukin-8 is the single most up-regulated gene in whole genome profiling of *H. Pylori* exposed gastric epithelial cells. BMC Microbiol. 12, 9. doi: 10.1186/1471-2180-12-9 22248188 PMC3292955

[B29] El Filaly H. Desterke C. Outlioua A. Badre W. Rabhi M. Karkouri M.. (2023). CXCL-8 as a signature of severe *helicobacter pylori* infection and a stimulator of stomach region-dependent immune response. Clin. Immunol. 252, 109648. doi: 10.1016/j.clim.2023.109648 37209806

[B30] El-Gebali S. Mistry J. Bateman A. Eddy S. R. Luciani A. Potter S. C.. (2019). The pfam protein families database in 2019. Nucleic Acids Res. 47, D427–D432. doi: 10.1093/nar/gky995 30357350 PMC6324024

[B31] El-Omar E. M. Rabkin C. S. Gammon M. D. Vaughan T. L. Risch H. A. Schoenberg J. B.. (2003). Increased risk of noncardia gastric cancer associated with proinflammatory cytokine gene polymorphisms. Gastroenterology 124, 1193–1201. doi: 10.1016/S0016-5085(03)00157-4 12730860

[B32] Faass L. Stein S. C. Hauke M. Gapp M. Albanese M. Josenhans C. (2021). Contribution of heptose metabolites and the cag pathogenicity island to the activation of monocytes/macrophages by *helicobacter pylori* . Front. Immunol. 12. doi: 10.3389/fimmu.2021.632154 PMC8174060 34093525

[B33] Gobert A. P. Wilson K. T. (2022). Induction and regulation of the innate immune response in *helicobacter pylori* infection. Cell Mol. Gastroenterol. Hepatol. 13, 1347–1363. doi: 10.1016/j.jcmgh.2022.01.022 35124288 PMC8933844

[B34] Gravina A. G. Zagari R. M. De Musis C. Romano L. Loguercio C. Romano M. (2018). *Helicobacter pylori* and extragastric diseases: A review. World J. Gastroenterol. 24, 3204–3221. doi: 10.3748/wjg.v24.i29.3204 30090002 PMC6079286

[B35] Harada A. Sekido N. Akahoshi T. Wada T. Mukaida N. Matsushima K. (1994). Essential involvement of interleukin-8 (IL-8) in acute inflammation. J. Leukoc. Biol. 56, 559–564. doi: 10.1002/jlb.56.5.559 7964163

[B36] Hu Y. Wan J.-H. Li X.-Y. Zhu Y. Graham D. Y. Lu N. H. (2017). Systematic review with meta-analysis: the global recurrence rate of *helicobacter pylori* . Alimentary Pharmacol. Ther. 46, 773–779. doi: 10.1111/apt.14319 28892184

[B37] Jiménez-Soto L. F. Haas R. (2016). The cagA toxin of *helicobacter pylori*: abundant production but relatively low amount translocated. Sci. Rep. 6, 23227. doi: 10.1038/srep23227 26983895 PMC4794710

[B38] Kao J. Y. Zhang M. Miller M. J. Mills J. C. Wang B. Liu M.. (2010). *Helicobacter pylori* immune escape is mediated by dendritic cell–induced treg skewing and th17 suppression in mice. Gastroenterology 138, 1046–1054. doi: 10.1053/j.gastro.2009.11.043 19931266 PMC2831148

[B39] Kelley L. A. Mezulis S. Yates C. M. Wass M. N. Sternberg M. J. (2015). The phyre2 web portal for protein modelling, prediction and analysis. Nat. Protoc. 10, 845–858. doi: 10.1038/nprot.2015.053 25950237 PMC5298202

[B40] Kim N. (2016). “Prevalence and transmission routes of *H. Pylori* ,” in Helicobacter pylori. Ed. Kim N. (Springer, Singapore), 3–19. doi: 10.1007/978-981-287-706-2

[B41] Kim M. K. Kim J. (2019). Properties of immature and mature dendritic cells: phenotype, morphology, phagocytosis, and migration. RSC Adv. 9, 11230–11238. doi: 10.1039/C9RA00818G 35520256 PMC9063012

[B42] Kranzer K. Eckhardt A. Aigner M. Knoll G. Deml L. Speth C.. (2004). Induction of maturation and cytokine release of human dendritic cells by *helicobacter pylori* . Infect. Immun. 72, 4416–4423. doi: 10.1128/iai.72.8.4416-4423.2004 PMC470701 15271898

[B43] Lamb A. Chen L. F. (2013). Role of the *helicobacter pylori*-induced inflammatory response in the development of gastric cancer. J. Cell Biochem. 114, 491–497. doi: 10.1002/jcb.24389 22961880 PMC3909030

[B44] Lee H. Choe G. Kim W. Kim H. Song J. Park K. (2006). Expression of lewis antigens and their precursors in gastric mucosa: relationship with *helicobacter pylori* infection and gastric carcinogenesis. J. Pathol. 209, 88–94. doi: 10.1002/(ISSN)1096-9896 16456898

[B45] Lepper P. M. Triantafilou M. Schumann C. Schneider E. M. (2005). Triantafilou, K. Lipopolysaccharides from *helicobacter pylori* can act as antagonists for toll-like receptor 4. Cell Microbiol. 7, 519–528. doi: 10.1111/cmi.2005.7.issue-4 15760452

[B46] Li H. Benghezal M. (2017). Crude preparation of lipopolysaccharide from *helicobacter pylori* for silver staining and western blot. Bio Protoc. 7, e2585. doi: 10.21769/BioProtoc.2585 PMC8438427 34595267

[B47] Li H. Liao T. Debowski A. W. Tang H. Nilsson H.-O. Stubbs K. A.. (2016). Lipopolysaccharide structure and biosynthesis in *helicobacter pylori* . Helicobacter 21, 445–461. doi: 10.1111/hel.12301 26934862

[B48] Li H. Marceau M. Yang T. Liao T. Tang X. Hu R.. (2019). East-asian *helicobacter pylori* strains synthesize heptan-deficient lipopolysaccharide. PloS Genet. 15, e1008497. doi: 10.1371/journal.pgen.1008497 31747390 PMC6892558

[B49] Lim S. H. Kwon J.-W. Kim N. Kim G. H. Kang J. M. Park M. J.. (2013). Prevalence and risk factors of *helicobacter pylori* infection in korea: nationwide multicenter study over 13 years. BMC Gastroenterol. 13, 104. doi: 10.1186/1471-230X-13-104 23800201 PMC3702482

[B50] Lin C. He H. Liu H. Li R. Chen Y. Qi Y.. (2019). Tumour-associated macrophages-derived CXCL8 determines immune evasion through autonomous PD-L1 expression in gastric cancer. Gut 68, 1764–1773. doi: 10.1136/gutjnl-2018-316324 30661053

[B51] Lina T. T. Alzahrani S. Gonzalez J. Pinchuk I. V. Beswick E. J. Reyes V. E. (2014). Immune evasion strategies used by *helicobacter pylori* . World J. Gastroenterol. 20, 12753–12766. doi: 10.3748/wjg.v20.i36.12753 25278676 PMC4177461

[B52] Liu J. Cao X. (2015). Regulatory dendritic cells in autoimmunity: A comprehensive review. J. Autoimmun 63, 1–12. doi: 10.1016/j.jaut.2015.07.011 26255250

[B53] Liu C.-H. Chen Z. Chen K. Liao F.-T. Chung C.-E. Liu X.. (2021). Lipopolysaccharide-mediated chronic inflammation promotes tobacco carcinogen-induced lung cancer and determines the efficacy of immunotherapy. Cancer Res. 81, 144–157. doi: 10.1158/0008-5472.CAN-20-1994 33122306 PMC7878420

[B54] Liu Q. Li A. Tian Y. Wu J. D. Liu Y. Li T.. (2016). The CXCL8-CXCR1/2 pathways in cancer. Cytokine Growth Factor Rev. 31, 61–71. doi: 10.1016/j.cytogfr.2016.08.002 27578214 PMC6142815

[B55] Lotz M. König T. Ménard S. Gütle D. Bogdan C. Hornef M. W. (2007). Cytokine-mediated control of lipopolysaccharide-induced activation of small intestinal epithelial cells. Immunology 122, 306–315. doi: 10.1111/j.1365-2567.2007.02639.x 17511808 PMC2266023

[B56] Lu H. Hsu P. I. Graham D. Y. Yamaoka Y. (2005). Duodenal ulcer promoting gene of *helicobacter pylori* . Gastroenterology 128, 833–848. doi: 10.1053/j.gastro.2005.01.009 15825067 PMC3130061

[B57] Lukáčová M. Barák I. Kazár J. (2008). Role of structural variations of polysaccharide antigens in the pathogenicity of gram-negative bacteria. Clin. Microbiol. Infect. 14, 200–206. doi: 10.1111/j.1469-0691.2007.01876.x 17986210

[B58] MaChado J. C. Figueiredo C. Canedo P. Pharoah P. Carvalho R. Nabais S.. (2003). A proinflammatory genetic profile increases the risk for chronic atrophic gastritis and gastric carcinoma. Gastroenterology 125, 364–371. doi: 10.1016/S0016-5085(03)00899-0 12891537

[B59] Malfertheiner P. Camargo M. C. El-Omar E. Liou J-M. Peek R. Schulz Z. (2023). *Helicobacter pylori* infection. Nat. Rev Dis Primers 9, 19–242. doi: 10.1038/s41572-023-00431-8 37081005 PMC11558793

[B60] Mandrell R. E. Apicella M. A. (1993). Lipo-oligosaccharides (LOS) of mucosal pathogens: molecular mimicry and host-modification of LOS. Immunobiology 187, 382–402. doi: 10.1016/S0171-2985(11)80352-9 8330904

[B61] Moulton K. D. Adewale A. P. Carol H. A. Mikami S. A. Dube D. H. (2020). Metabolic glycan labeling-based screen to identify bacterial glycosylation genes. ACS Infect. Dis. 6, 3247–3259. doi: 10.1021/acsinfecdis.0c00612 33186014 PMC7808405

[B62] Moya D. A. Crissinger K. D. (2012). *Helicobacter pylori* persistence in children: distinguishing inadequate treatment, resistant organisms, and reinfection. Curr. Gastroenterol. Rep. 14, 236–242. doi: 10.1007/s11894-012-0251-y 22350943

[B63] Olivera-Severo D. Uberti A. F. Marques M. S. Pinto M. T. Gomez-Lazaro M. Figueiredo C.. (2017). A new role for *helicobacter pylori* urease: contributions to angiogenesis. Front. Microbiol. 8. doi: 10.3389/fmicb.2017.01883 PMC5623709 29021786

[B64] Outlioua A. Badre W. Desterke C. Echarki Z. El Hammani N. Rabhi M.. (2020). Gastric IL-1β, IL-8, and IL-17A expression in moroccan patients infected with *helicobacter pylori* may be a predictive signature of severe pathological stages. Cytokine 126, 154893. doi: 10.1016/j.cyto.2019.154893 31877554

[B65] Pachathundikandi S. K. Backert S. (2016). Differential expression of interleukin 1β During *helicobacter pylori* infection of toll-like receptor 2 (TLR2)– and TLR10-expressing HEK293 cell lines. J. Infect. Dis. 214, 166–167. doi: 10.1093/infdis/jiw154 27091910

[B66] Pachathundikandi S. K. Lind J. Tegtmeyer N. El-Omar E. M. Backert S. (2015). Interplay of the gastric pathogen *helicobacter pylori* with toll-like receptors. BioMed. Res. Int. 2015, e192420. doi: 10.1155/2015/192420 PMC4402485 25945326

[B67] Pérez-Pérez G. I. Shepherd V. L. Morrow J. D. Blaser M. J. (1995). Activation of human THP-1 cells and rat bone marrow-Derived macrophages by *helicobacter pylori* lipopolysaccharide. Infect. Immun. 63, 1183–1187. doi: 10.1128/iai.63.4.1183-1187.1995 7890370 PMC173132

[B68] Pfannkuch L. Hurwitz R. Traulsen J. Sigulla J. Poeschke M. Matzner L.. (2019). ADP heptose, a novel pathogen-associated molecular pattern identified in *helicobacter pylori* . FASEB J. 33, 9087–9099. doi: 10.1096/fj.201802555R 31075211 PMC6662969

[B69] Posselt G. Crabtree J. E. Wessler S. (2017). Proteolysis in *helicobacter pylori*-induced gastric cancer. Toxins 9, 134. doi: 10.3390/toxins9040134 28398251 PMC5408208

[B70] Prado Acosta M. Lepenies B. (2019). Bacterial glycans and their interactions with lectins in the innate immune system. Biochem. Soc. Trans. 47, 1569–1579. doi: 10.1042/BST20170410 31724699

[B71] Qi W.-Q. Zhang Q. Wang J.-B. (2020). CXCL8 is a potential biomarker for predicting disease progression in gastric carcinoma. Transl. Cancer Res. 9, 1053–1062. doi: 10.21037/tcr 35117450 PMC8797801

[B72] Rad R. Gerhard M. Lang R. Schöniger M. Rösch T. Schepp W.. (2002). The *helicobacter pylori* blood group antigen-binding adhesin facilitates bacterial colonization and augments a nonspecific immune response. J. Immunol. 168, 3033–3041. doi: 10.4049/jimmunol.168.6.3033 11884476

[B73] Reshetnyak V. I. Reshetnyak T. M. (2017). Significance of dormant forms of *helicobacter pylori* in ulcerogenesis. World J. Gastroenterol. 23, 4867–4878. doi: 10.3748/wjg.v23.i27.4867 28785141 PMC5526757

[B74] Robinson K. Argent R. H. Atherton J. C. (2007). The inflammatory and immune response to *helicobacter pylori* infection. Best Pract. Res. Clin. Gastroenterol. 21, 237–259. doi: 10.1016/j.bpg.2007.01.001 17382275

[B75] Schirm M. Soo E. C. Aubry A. J. Austin J. Thibault P. Logan S. M. (2003). Structural, genetic and functional characterization of the flagellin glycosylation process in. Helicobacter Pylori. Mol. Microbiol. 48, 1579–1592. doi: 10.1046/j.1365-2958.2003.03527.x 12791140

[B76] Sedighzadeh S. S. Galehdari H. Tabandeh M. R. Roohbakhsh A. Shamsara M. (2020). The LPS-treated human gastric cancer cells (AGS) show a significant higher tendency to proliferation, inflammation and cannabinoid receptor 1 expression. Jentashapir J. Cell Mol. Biol. 11, e111280. doi: 10.5812/jjcmb

[B77] Shi Y. Zheng H. Wang M. Ding S. (2022). Influence of *helicobacter pylori* infection on PD-1/PD-L1 blockade therapy needs more attention. Helicobacter 27, e12878. doi: 10.1111/hel.12878 35112435

[B78] Simoons-Smit I. M. Appelmelk B. J. Verboom T. Negrini R. Penner J. L. Aspinall G. O.. (1996). Typing of *helicobacter pylori* with monoclonal antibodies against lewis antigens in lipopolysaccharide. J. Clin. Microbiol. 34, 2196–2200. doi: 10.1128/jcm.34.9.2196-2200.1996 8862584 PMC229216

[B79] Smedley J. G. Jewell E. Roguskie J. Horzempa J. Syboldt A. Stolz D. B.. (2005). Influence of pilin glycosylation on *pseudomonas aeruginosa* 1244 pilus function. Infect. Immun. 73, 7922–7931. doi: 10.1128/IAI.73.12.7922-7931.2005 16299283 PMC1307089

[B80] Smith S. M. (2014). Role of toll-like receptors in *helicobacter pylori* infection and immunity. World J. Gastrointest Pathophysiol 5, 133–146. doi: 10.4291/wjgp.v5.i3.133 25133016 PMC4133513

[B81] Stefano K. Marco M. Federica G. Laura B. Barbara B. Gioacchino L.. (2018). *Helicobacter pylori*, transmission routes and recurrence of infection: state of the art. Acta BioMed. 89, 72–76. doi: 10.23750/abm.v89i8-S.7947 PMC6502203 30561421

[B82] Suerbaum S. Michetti P. (2002). *Helicobacter pylori* infection. New Eng. J. Med. 347, 1175–1186. doi: 10.1056/NEJMra020542 12374879

[B83] Tanaka H. Yoshida M. Nishiumi S. Ohnishi N. Kobayashi K. Yamamoto K.. (2010). The cagA protein of *helicobacter pylori* suppresses the functions of dendritic cell in mice. Arch. Biochem. Biophys. 498, 35–42. doi: 10.1016/j.abb.2010.03.021 20363211

[B84] Teng K.-W. Hsieh K.-S. Hung J.-S. Wang C.-J. Liao E.-C. Chen P.-C.. (2022). *Helicobacter pylori* employs a general protein glycosylation system for the modification of outer membrane adhesins. Gut Microbes 14, 2130650. doi: 10.1080/19490976.2022.2130650 36206406 PMC9553153

[B85] Thung I. Aramin H. Vavinskaya V. Gupta S. Park J. Y. Crowe S. E.. (2016). Review article: the global emergence of *helicobacter pylori* antibiotic resistance. Alimentary Pharm. Ther. 43, 514–533. doi: 10.1111/apt.2016.43.issue-4 PMC5064663 26694080

[B86] Tra V. N. Dube D. H. (2014). Glycans in pathogenic bacteria – potential for targeted covalent therapeutics and imaging agents. Chem. Commun. 50, 4659–4673. doi: 10.1039/C4CC00660G PMC4049282 24647371

[B87] Tshibangu-Kabamba E. Yamaoka Y. (2021). *Helicobacter pylori* infection and antibiotic resistance - from biology to clinical implications. Nat. Rev. Gastroenterol. Hepatol. 18, 613–629. doi: 10.1038/s41575-021-00449-x 34002081

[B88] van der Woude M. W. Bäumler A. J. (2004). Phase and antigenic variation in bacteria. Clin. Microbiol. Rev. 17, 581–611. doi: 10.1128/CMR.17.3.581-611.2004 15258095 PMC452554

[B89] Vannini A. Roncarati D. Spinsanti M. Scarlato V. Danielli A. (2014). In depth analysis of the *helicobacter pylori* cag pathogenicity island transcriptional responses. PloS One 9, e98416. doi: 10.1371/journal.pone.0098416 24892739 PMC4043881

[B90] Wang G. Ge Z. Rasko D. A. Taylor D. E. (2000). Lewis antigens in *helicobacter pylori*: biosynthesis and phase variation. Mol. Microbiol. 36, 1187–1196. doi: 10.1046/j.1365-2958.2000.01934.x 10931272

[B91] Waugh D. J. J. Wilson C. (2008). The interleukin-8 pathway in cancer. Clin. Cancer Res. 14, 6735–6741. doi: 10.1158/1078-0432.CCR-07-4843 18980965

[B92] White J. R. Winter J. A. (2015). Robinson, K. Differential inflammatory response to *helicobacter pylori* infection: etiology and clinical outcomes. J. Inflammation Res. 8, 137–147. doi: 10.2147/JIR.S64888 PMC4540215 26316793

[B93] Williams D. A. Pradhan K. Paul A. Olin I. R. Tuck O. T. Moulton K. D.. (2020). Metabolic inhibitors of bacterial glycan biosynthesis. Chem. Sci. 11, 1761–1774. doi: 10.1039/C9SC05955E 34123271 PMC8148367

[B94] Wirth H. P. Yang M. Peek R. M. Höök-Nikanne J. Fried M. Blaser M. J. (1999). Phenotypic diversity in lewis expression of *helicobacter pylori* isolates from the same host. J. Lab. Clin. Med. 133, 488–500. doi: 10.1016/S0022-2143(99)90026-4 10235132

[B95] Yamaoka Y. Kikuchi S. El–Zimaity H. M. T. Gutierrez O. Osato M. S. Graham D. Y. (2002). Importance of *helicobacter pylori* oipA in clinical presentation, gastric inflammation, and mucosal interleukin 8 production. Gastroenterology 123, 414–424. doi: 10.1053/gast.2002.34781 12145793

[B96] Yoshimura T. Tomita T. Dixon M. F. Axon A. T. R. Robinson P. A. Crabtree J. E. (2002). ADAMs (A disintegrin and metalloproteinase) messenger RNA expression in *helicobacter pylori*—Infected, normal, and neoplastic gastric mucosa. J. Infect. Dis. 185, 332–340. doi: 10.1086/338191 11807715

