# Supplementary material for: Helicobacter pylori glycan biosynthesis modulates host immune cell recognition and response
Source: Front Cell Infect Microbiol. 2024 Mar 20;14:1377077. doi: 10.3389/fcimb.2024.1377077 (PMC10987845; doi:10.3389/fcimb.2024.1377077)
Supplement: Supplementary file 1 [file DataSheet_1.pdf]

Supplementary Material

Table of Contents

1 Supplementary Methods……………………………………….……………….……..……….....*s*-2

General……………………………………………..………………….……….……..……...*s*-2
H. pylori growth conditions and strains used in this study………...……….….……..…....*s*-2
Bioinformatics analysis to identify a putative oligosaccharyltransferase gene……….……..*s*-2
Generation of insertionally inactivated *H. pylori* glycosylation mutants…………………...*s*-3
Detection of glycoprotein biosynthesis in *H. pylori* mutants……...……...………..…..........*s*-3
H. pylori growth prior to challenging gastric cells or immature dendritic cells.……. …......*s*-4
Culturing gastric epithelial cells…..……………………………………………..……......*s*-4
Culturing immature dendritic cells…..…………………….…………………….………......*s*-5
H. pylori and gastric epithelial cell co-cultures.……………………………….……........*s*-5
Detection of cytokines via ELISA...………………………………………. …....…....*s*-5
H. pylori and immature dendritic cell co-cultures.….. …………………………….. ….......*s*-6
Flow cytometry analysis of immature dendritic cells………………………………….……..*s*-6
Anti-Lewis Y immunoblotting.…..…………………… ……………..…...…………....*s*-6
Crude LPS extraction and visualization………………………………………….…..…s-7

2 Supplementary Tables…………………..……………….………….…….……….…….…*s*-8

Table S1………………………………………………………………..………….....*s*-8
Table S2. ………………………………...……………………….…………….……...*s*-9
Table S3. ………………………………...……………………….…………….……...*s*-10

3 Supplementary Figures…………………..………………..………..………………....…*s*-11

Figure S1…………….………………………………………………..….……………...*s*-11
Figure S2………………..……………………………………………..………………...*s*-12
Figure S3…………….………………………………………………..….……………...*s*-13

4 Supplementary References…..………………………………………………………......*s*-14

1 Supplementary Methods

Materials

Tissue culture reagents, tissue culture plates, and ELISA kits were purchased from ThermoFisher Scientific (Waltham, MA), MilliporeSigma (Burlington, MA), R&D Systems (Minneapolis, MN), and USA Scientific (Ocala, FL). Tissue culture and bacterial work were completed using standard aseptic techniques.

H. pylori growth conditions and strains used in this study

H. pylori strain G27 was a gift from Manuel Amieva (Stanford University). Wildtype (WT) G27 *H. pylori* were grown on Horse Blood Agar plates (HBA) made from 4% Columbia agar base, 5% horse blood, 10 μg/mL of vancomycin, 5 μg/mL cefsulodin, 0.3 μg/mL polymyxin B, 5 μg/mL trimethoprim, and 8 μg/mL amphotericin B. *H. pylori* were grown at 37 °C in 14% CO_2. Glycosylation mutants of *H. pylori* bearing a chloramphenicol acetyl transferase (*cat*) cassette were grown on HBA plates supplemented with 34 μg/mL of chloramphenicol. Different freezer lots were used in each experiment. The glycosylation mutants Δ*579* and Δ*580* were previously constructed and characterized.[27] *H. pylori* G27 mutants Δ*wzk*, Δ*waaL*, and Δ*1179* were constructed in this study. See Table S1 for a list of genes explored in this study and their orthologs in the reference strain 26695, and Table S2 for the list of strains used in this study, their genotype, and notes.

Bioinformatics analysis to identify a putative oligosaccharyltransferase gene

With the goal of identifying a putative oligosaccharyltransferase gene involved in *H. pylori's* general protein glycosylation system, a bioinformatics analysis of *H. pylori* G27 was conducted. Briefly, whole genome alignment was performed using Mauve (version 1.1.3) whole genome alignment on Geneious Prime 2020[1] to identify genes encoding glycosyltransferases that were conserved across other *H. pylori* genomes (G27, 26695, J99, and P12). The G27 genome was analyzed for open reading frames using Glimmer[2] on the KBase Server[3] with the RAST (RASTtk – v1.073) pipeline[4], which was preloaded onto the KBase server and run with default parameters as "Annotate Microbial Genome." Genetic analysis of the G27 genomes was performed using Geneious Prime 2020, and the comparison of genes was done using BLAST[5]. Domains were called using HMMER[6] search against the G27 genomes using the PFam HMM 32.0 database[7]. For open reading frames that did not have previous biochemical characterization, homology was analyzed using the PHYRE2 recognition server[8]. To identify individual genes for study, the HMMER assigned Pfam domains were used to find open reading frames (ORFs) encoding genes with glycosyltransferase domains that lacked biochemical characterization[7]. These genes were selected and annotated using Phyre2[8]. The list of possible genes was further reduced through removal of ORFs containing genes well described in other functions, and the choices were ranked based on the likelihood they were involved in sugar metabolism. This approach led to the identification of *HpG27_1179* as a putative oligosaccharyltransferase that may play a role in *H. pylori*'s general protein glycosylation system. *HpG27_1179* was annotated to encode a putative glycosyltransferase 39 family member by the automatic annotation from NCBI and was annotated as a Pfam - dolichyl-phosphate-mannose-protein mannosyltransferase[7]. When the protein encoded by *HpG27_1179* was modeled on Phyre2[8], it exhibited significant homology (multiple models with greater than 99% confidence made by Phyre2[8]) to enzymes involved in the transfer of glycans onto proteins in other systems including a *Campylobacter lari* oligosaccharide transferase PglB[9] (PDB:3RCE) and multiple dolichyl-phosphate-

mannose-protein mannosyltransferases, such as the eukaryotic yeast *Saccharomyces cerevisiae*'s Pmt1[10] (PDB:6P25).

Generation of insertionally inactivated H. pylori glycosylation mutants

Gibson assembly was used to generate linear DNA fragments for insertional inactivation of target genes with a chloramphenicol acetyl transferase (*cat*) cassette[11]. Geneious Prime 2020 Gibson Assembly Tool was used to generate primers used for amplification; primers were chosen such that they had 15-20 base pairs to anneal to the template, along with an overlap sequence. Primers used for PCR and sequencing are in Table S3. Briefly, *HpG27_389* (*waaL*), *HpG27_1153* (*wzk*), and *HpG27_1179* from wildtype *H. pylori* strain G27 were amplified and constructed to contain *cat* insertions via Gibson assembly using the primers listed in Table S3. *HpG27_389* (*waaL*) and *HPG27_1153* (*wzk*) were selected for insertional inactivation because of their established roles acting as the O-antigen ligase and O-antigen flippase, respectively, in LPS biosynthesis in *H. pylori*. *HpG27_1179* was identified via bioinformatics analysis as a putative oligosaccharyltransferase in *H. pylori* and insertionally inactivated to probe its possible role in glycoprotein biosynthesis.

For each insert made, three fragments were amplified: 1) the first gene fragment (referred to as WaaL.1, Wzk.1, or 1179.1) with the forward and reverse (with overlap onto *cat*) primers, 2) the second gene fragment (referred to as WaaL.2, Wzk.2, or 1179.2), with the forward (with overlap onto *cat*) and reverse primers, and 3) the *cat* cassette from G27*ΔHpG27_580* gDNA[12], with the forward and reverse primers overlapping with the first and second gene fragments, respectively. PCR was performed using OneTaq 2x Master Mix (New England Biolabs), with *H. pylori* G27 wild type genomic DNA and G27*ΔHpG27_580*[12] gDNA used as template to amplify target genes and the *cat* cassette, respectively. Amplification of the three fragments was confirmed by electrophoresis, and PCR products from each reaction were isolated following electrophoresis on 1% agarose (120 V, TriDye™ 1 kb Plus DNA Ladder (New England Biolabs, Ipswich, MA), Ethidium bromide stain) using QIAquick Gel Extraction Kit (QIAGEN, Germantown, MD). DNA inserts were assembled by attaching the first gene fragment, the second gene fragment, and the *cat* cassette using Gibson Assembly 2x Master Mix (New England Biolabs) at 50 °C for 15 minutes. Assembly of the insertionally inactivated genes was confirmed by gel electrophoresis, and the product was purified using QIAquick Gel Extraction Kit (QIAGEN) into ddH$_2$O. The resulting linear DNA was transformed into wildtype strain G27 through natural transformation using the patch method, and gene interruption was autonomously completed by homologous recombination. The successful insertion of the resistance cassette in mutant strains selected on chloramphenicol/HBA agar was confirmed by polymerase chain reaction (PCR) analysis of genomic DNA from selected mutants. Diagnostic PCR revealed the absence of the wildtype target gene (*wzk*, *waaL*, and *1179*) and the presence of the inactivated target gene (*wzk::cat*, *waaL::cat*, and *1179::cat*) in genomic DNA isolated from Δ*wzk*, Δ*waaL*, and Δ*1179*, respectively.

Detection of glycoprotein biosynthesis in H. pylori mutants

A previously reported metabolic glycan labeling strategy was used to detect glycoprotein biosynthesis in wildtype *H. pylori* and newly constructed glycosylation mutants Δ*wzk*, Δ*waaL*, and Δ*1179*. Briefly, *H. pylori* strains were grown in Brucella Broth (with 34 µg/ml Chlor for mutants) containing 1 mM Ac$_4$GlcNAz to metabolically label newly synthesized glycoproteins with azides. As a negative control, wildtype *H. pylori* was grown in Brucella Broth containing 1.0 mM Ac$_4$GlcNAc, an azide-free version of the sugar. Cells were grown in microaerophilic conditions for 4 days, then

pelleted and washed with PBS three times. Cells were resuspended in *H. pylori* lysis buffer with protease inhibitor (20 mM Tris-HCl, pH 7.4, 1% Igepal, 150 mM NaCl, 1 mM EDTA, Protease inhibitor (MilliporeSigma)) and were frozen for 30 minutes to lyse cells. The protein concentration of lysates was determined using a Lowry assay, and lysate concentrations were standardized to 2.5 mg/ml and reacted with a final concentration of 250 µM Phos-FLAG[13] overnight at room temperature to label azides with a FLAG epitope. Following overnight reaction with Phos-FLAG, treated samples were electrophoresed via SDS-PAGE on a Mini-PROTEAN TGX Stain-Free Precast 12% acrylamide gel with 4% stacking layer (Bio-Rad). Gels were electrophoresed with a Mini-PROTEAN Gel electrophoresis system (Bio-Rad) at 100 V for 5 minutes and 200 V for 40 minutes in SDS running buffer (3 g/L Tris-Base, 14.4 g/L glycine, 1 g/L SDS, ddH_2O). To evaluate protein loading, the gel was submerged in Coomassie stain (45% ddH_2O, 45% methanol, 10% glacial acetic acid, 0.25% Coomassie brilliant blue) and microwaved for 30 seconds, then stained for 10 minutes. The gel was then destained with Coomassie destain (50% deionized water, 40% methanol, 10% acetic acid) until bands were visible. For azide detection, western blot analysis was with anti-FLAG antibody was conducted. The protein was transferred onto a nitrocellulose membrane (Amersham, Burlington, MA) in a transfer cassette in cold transfer buffer by electrophoresis at 100 V for 45 minutes in a Mini-PROTEAN Gel electrophoresis system (Bio-Rad). The membrane was blocked in 5% nonfat milk in tris buffered saline with Tween-20 (TBS-T) (5 mM Tris-HCl, 0.05% Tween-20 (Fisher Scientific)) for 60 minutes. After three 5-minute washes in TBS-T, the membrane was incubated with anti-FLAG M2-HRP[14] (MilliporeSigma; diluted 1:1000 in TBS-T) for one hour. After another three 5-minute washes in TBS-T, the membrane was incubated with luminol/peroxidase reagent (SuperSignal West Pico Plus Chemiluminescent Substrate, ThermoFisher) for one minute and documented on a Genesys G-Box (Genesys).

H. pylori growth prior to challenging gastric cells or immature dendritic cells

Prior to each co-culture experiment, wildtype *H. pylori* were grown for four days, and mutants were grown for four or five days at 37 °C in 14% CO_2 until a full lawn of bacteria growth was observed. The bacteria were removed from the plates and transferred into 3.5 mL of phosphate-buffered saline to measure an OD_{600} of 0.10 – 0.14, which corresponds to a concentration of (1.0– 1.4) x 10^8 cells per mL for cocultures with adenocarcinoma-derived gastric epithelial (AGS) cells. For cocultures with immature dendritic cells, bacteria were diluted to a concentration of 4.7 x 10^7 cells per mL. The bacterial cells were pelleted and resuspended in the same volume of mammalian cell media, either Ham's F12 Glutamax with 10% fetal bovine serum (FBS) for gastric cell coculture, or RPMI 1640 with glutamine with 10% FBS for dendritic cell coculture.

Culturing gastric epithelial cells

Adenocarcinoma-derived gastric epithelial cells termed AGS cells (ATCC Number: CRL-1739) were stored in liquid nitrogen. After thawing the cryogen tube in a 37 °C water bath, the AGS cells were transferred into fresh AGS media (Ham's F12 Glutamax and 10% FBS) in a T-75 flask. The cells were incubated at 37 °C with 5% CO_2 for 48 hours upon initial seeding. The AGS cells were subsequently passaged upon reaching 80-90% confluency and split at a 1:10 dilution every 3-4 days for the duration of the experiment to keep cells healthily propagated and deter over-confluency. To passage AGS cells, old AGS media and dead non-adherent cells were removed from the T-75 flask. Cells in the flask were washed with PBS, then treated with 0.25% Trypsin and EDTA for 5 minutes at 37 °C and 5% CO_2. New AGS media was added to the flask to quench the trypsin, and cells were

resuspended to a concentration of roughly 3 x 10^5 cells/mL (1:10 or 1:20 dilution) depending on the length of time before the next experiment. New AGS media was added accordingly.

Culturing immature dendritic cells

Human leukemia-derived THP-1 monocytes were removed from liquid nitrogen and thawed before immediately resuspending in 10 mL of warm RPMI 1640 with glutamine with 20% FBS. The cells were then centrifuged at 100 x g for 10 min, and the pellet was resuspended in 10 mL of the media again before adding to T-25 flasks to grow in 37 °C and 5% CO_2. Every few days, cell viability was checked and cells were counted. Cell concentrations above 1 x 10^6 cells per mL were diluted 1:3 with more RPMI media and seeded in 20 mL of media in T-75 flasks. Cells were subsequently diluted at a split of 1:3 – 1:5 with fresh RPMI media every 3-4 days to prevent over-crowding.

H. pylori and gastric epithelial cell cocultures

Approximately 5 x 10^5 gastric cells/mL were seeded into 6-well tissue culture plates in 1 mL media. Gastric epithelial cells were grown for 48 hours, to around 90% confluency, at 37 °C with 5% CO_2. After this incubation, 1 mL of the *H. pylori* suspension in AGS media (or media alone for control samples) was added into each well at a concentration of 1.0 x 10^8 – 1.4 x 10^8 bacteria/mL. For heat-killed challenges, bacteria were heated for 10 min at 70 °C before being added to gastric cell culture as described. The coculture was then incubated at 37 °C with 5% CO_2 for 3 or 6 hours, as indicated below, before harvesting supernatants for further analysis.

Detection of cytokines via ELISA

After 3- and 6-hour cocultures for AGS cells or 24-hour cocultures for immature dendritic cells, the supernatants of the cells in coculture were harvested and centrifuged at 15,000 x *g* to remove any cells. The supernatants were then isolated and stored in a -80 °C freezer until further analyses. A Human CXCL-8 (IL-8), IL-10, TNF-α, IL-6, or IL-1β DuoSet Enzyme Linked Immunoassay (ELISA) kit (R&D Systems, Minneapolis, MN) was utilized to detect relative concentrations of CXCL-8, IL-10, TNF-α, IL-6, or IL-1β, respectively, in the various *H. pylori* and gastric cell cocultures. All ELISA assays were conducted according to the manufacturer's instructions. First, an anti-human CXCL-8, IL-10, TNF-α, IL-6, or IL-1β, respectively, capture antibody was added in working concentration (1:120) to a 96-well plate for an overnight incubation at room temperature. The following day, after washing with wash buffer diluted in ddH_2O (1:25), the plate was blocked with proprietary blocking buffer (reagent diluent at 1:10 dilution of the working reagent concentration) and incubated for an hour at room temperature. Upon repeating the washing step, samples (thawed coculture supernatants) were added to the plate, as well as standards diluted in reagent diluent, at a volume of 100 µL per 96-well. After incubating for 2 hours at room temperature, the plate was washed as before, and biotinylated goat anti-human CXCL-8, IL-10, TNF-α, IL-6, or IL-1β, respectively, detection antibodies (1:60) were added to each well for 2 hours at room temperature. The plate was washed again and treated with streptavidin conjugated to horseradish-peroxidase (1:40) for 20 min at room temperature. Next, the plate was treated with equal parts H_2O_2 and the substrate tetramethylbenzidine for 20 min to initiate color change, followed by the addition of H_2SO_4 to stop the enzymatic reaction. Finally, absorbances in the ELISA plate were measured by a SpectroStar Nano microplate reader (BMG Labtech, Ortenberg, Germany) at 450 nm wavelength. All cytokine concentration data was derived from an 8-point standard curve created using recombinant human CXCL-8, IL-10, TNF-α, IL-6, or IL-1β, respectively, standards included in the DuoSet kits.

H. pylori and immature dendritic cell cocultures

To differentiate the THP-1 monocytes into immature dendritic cells (iDCs), the THP-1 monocytes were incubated at a concentration of 2 x 10^5 cells/mL for 5 days with human recombinant cytokines IL-4 (100 ng = 1500 IU/mL) and GM-CSF (100 ng = 1500 IU/mL) to allow them to mature into iDCs and adhere to the plate for further analysis. After 5 days of incubation, cells were centrifuged and resuspended in half of the original media to increase the concentration by two. DC suspension (0.5 mL) was added to wells in a 24-well plate, at a final concentration of 2 x 10^5 cells per well. *H. pylori* resuspended in RPMI media with 10% FBS at a concentration of 2 x 10^7 cells/mL were then added to the plate at an equal volume. The final ratio of iDCs to bacteria was 1:100 in each well. Immature dendritic cell and *H. pylori* coculture were incubated for approximately 24 hours at 37 °C with 5% CO_2 before harvesting cell culture for further analysis.

Flow cytometry analysis of immature dendritic cells

After coculturing the iDCs with *H. pylori* for approximately 24 hours, the cell cultures were transferred to microcentrifuge tubes, centrifuged at 250 x g for 10 minutes, and resuspended of RPMI media with 10% FBS with 1:40 dilution of both FITC conjugated CD80 and PE conjugated CD86 goat anti-human antibodies for 30 min on ice (BD Biosciences, Franklin Lakes, NJ). The cells were then washed three times with PBS, spinning at 250 x g for 10 min, and resuspended an additional time in PBS for flow cytometry analysis. CD80 and CD86 expression levels were measured using a BD Accuri C6 Flow Cytometer and analyzed using FlowJo software (TreeStar, Ashland, OR). BD CS&T RUO beads were used to standardize fluorescence intensity.

For each sample, 10,000 dendritic cells were counted. Single cells were first gated by plotting Forward Scatter Height (FSC-H) versus Forward Scatter Area (FSC-A) and selecting cells that fell along the linear curve. Wildtype *H. pylori* was analyzed via flow cytometry at 10,000 counts to determine bacterial cell size. From this, within the single cell population of all data points, *H. pylori* were gated out based on size to isolate single iDCs for each sample. Relative fluorescence was quantified using this gated population of cells for each sample.

Anti-Lewis Y immunoblotting

In parallel to immature dendritic cell challenge, a same-day plating of the same lot of bacterial cells was used to analyze Lewis Y produced by bacterial cells. Cells were resuspended in *H. pylori* lysis buffer with protease inhibitor (20 mM Tris-HCl, pH 7.4, 1% Igepal, 150 mM NaCl, 1 mM EDTA, Protease inhibitor (MilliporeSigma)) and were frozen for 30 minutes to lyse cells. The protein concentration of lysates was determined using a Lowry assay, and lysate concentrations were standardized to 2.5 mg/ml. Samples were electrophoresed via SDS-PAGE on a Mini-PROTEAN TGX Stain-Free Precast 12% acrylamide gel with 4% stacking layer (Bio-Rad). Coomassie stain was used to evaluate protein loading. To evaluate protein loading, the gel was submerged in Coomassie stain (45% ddH_2O, 45% methanol, 10% glacial acetic acid, 0.25% Coomassie brilliant blue) and microwaved for 30 seconds, then stained for 10 minutes. The gel was then destained with Coomassie destain (50% deionized water, 40% methanol, 10% acetic acid) until bands were visible. To evaluate Lewis Y expression, an electrophoresed gel was transferred to a nitrocellulose membrane (Amersham) via electrophoresis at 100V for 60 minutes in transfer buffer. The nitrocellulose membrane was blocked in 5% nonfat milk solution (5% w/v in 0.05% TBS-Tween (pH 7.4, 5mM Tris-HCl, 0.05% Tween-20 (Fisher Scientific)) for 1 hour at room temperature, then washed twice for

five minutes with 0.05% TBS-Tween (TBST). The nitrocellulose membrane was then incubated with anti-Lewis Y antibody (Abcam, Waltham, MA) diluted in blocking solution, then washed twice for five minutes with 0.05% TBST. The nitrocellulose membrane was next incubated with 1:3000 Anti-Mouse IgM HRP (Southern Biotech, Birmingham, AL) diluted in blocking solution for one hour, then washed five times for five minutes each in 0.05% TBST. The nitrocellulose membrane was then treated with 1:1 SuperSignal® West Pico Luminol Enhancer Solution/SuperSignal® West Pico Stable Peroxide Solution (ThermoScientific) chemiluminescent substrate to visualize the HRP-tagged antibody using a Syngene G box (Cambridge, UK).

Crude LPS extraction and visualization

In parallel to immature dendritic cell challenge, a same-day plating of the same lot of bacterial cells was used to analyze LPS produced by bacterial cells. Bacterial lysates were diluted in 100 µL of LPS lysis buffer (10% SDS, 4% β-mercaptoethanol, 0.06 mg/mL Bromophenol blue, 10% glycerol, 75% 1M Tris-HCl (pH 6.8))[41] and incubated at 100 °C for 10 minutes. After cooling to room temperature, the samples were treated with 5 µL of 20 mg/mL Proteinase K (New England Biolabs, #P8102S) at 55 °C overnight in a water bath. To visualize LPS, 20 µL of samples were added to gels (4-20% Tris-HCl SDS-PAGE gels). CandyCane Glycoprotein Molecular Weight standard (Fisher Scientific) and 250 µg/mL lipopolysaccharide from *E. coli* serotype 055:B5 (Fisher Scientific) were added as well as control and size markers. Gels were electrophoresed at 200 V for 50 minutes in 1X SDS running buffer (ddH_2O, 3.47 mM SDS, 24.71 mM Tris base, 191.95 mM glycine) in a Mini-PROTEAN Tetra Cell (Bio-Rad, Hercules, CA). Gels were stained according to the ProQ Emerald 300 Lipopolysaccharide Gel Stain Kit (Thermo Fisher Scientific) manufacturer's instructions. Stained gels were then visualized with a UVP BioDoc-It® Imaging System (Upland, CA).

2 Supplementary Tables

Table S1. List of G27 genes explored this study, with names of orthologs in the reference strain 26695 provided.

**26695 ORF**	**G27 ORF**	**Activity**	**References**
HP0619 *(JHP0562/0563)*	*HPG27_579/580*	β-1,3-galactosyltransferase	[15]
HP1206	*HPG27_389*	Wzk (flippase)	[16, 17]
HP1039	*HPG27_1153*	WaaL (ligase)	[16, 17]
HP1235	*HPG27_1179*	Putative OST	This study

Table S2. List of strains used in this study.

Strain name	Genotype	Notes
H. pylori G27	wildtype	sequenced[18]
Δ579	*579::cat*	targeted insertion[19]
Δ580	*580::cat*	targeted insertion[19]
Δwzk	*wzk::cat*	targeted insertion, this study
ΔwaaL	*waaL::cat*	targeted insertion, this study
Δ1179	*1179::cat*	targeted insertion, this study

Table S3. Primers used in the Gibson Assembly reactions to insert the chloramphenicol acetyl transferase (*cat*) resistance cassette into target genes.

Primer	*Sequence**
WaaL.1F	GTGTTGAAAGAGCGTTTGAAAGC
WaaL.1R- CAT	CCGTAGTAGCAATAAATTGTAGCGGAAAGTTTGGC
CAT-F- WaaL.1	**ACAATTTATT**GCTACTACGGCAGGCTACTA
CAT-R - WaaL.2	**TTGGCTCTA**TATCAGTGCGACAAACTGGGA
WaaL.2F - CAT	GCACTGATATAGAGCCAAAGATTTGATTCTTTGT
WaaL.2R	CTAAAAAGCGCTTTTATCCT
Wzk.1F	ATAAAATTTCTACTTTAAAATACTTTTTGCGT
Wzk.1R- CAT	CGTAGTAGCCATAAAACATCCTGAACAAATAAATCCCC
CAT-F- Wzk.1	**TGTTTTATG**GCTACTACGGCAGGCTACTA
CAT-R- Wzk.2	**AAAAATCATA**TATCAGTGCGACAAACTGGGA
Wzk.2F - CAT	CGCACTGATATATGATTTTTTATGCGAGCATGAGGG
Wzk.2R	TTAGCCAAGATTGTCTTTGTGTTGG
1179.1F	ATGCAGCTAAGCCCCTTACA
1179.1R - CAT	CGTAGTAGCAAAAACATGTTAATGATATGAGCGA
CAT-F - 1179.1	**CATGTTTTT**GCTACTACGGCAGGCTACTA
CAT-R - 1179.2	**ATTAAAAGA**TTATCAGTGCGACAAACTGGGA
1179.2F - CAT	CGCACTGATAATCTTTTAATGCTCACTGAAACGCT
1179.2R	TTAATGGCGCACCAAACTATAGG

* Bold indicates cassette primer overlap with gene fragments; underline indicates gene fragment primer overlap with cassette.

3 Supplementary Figures

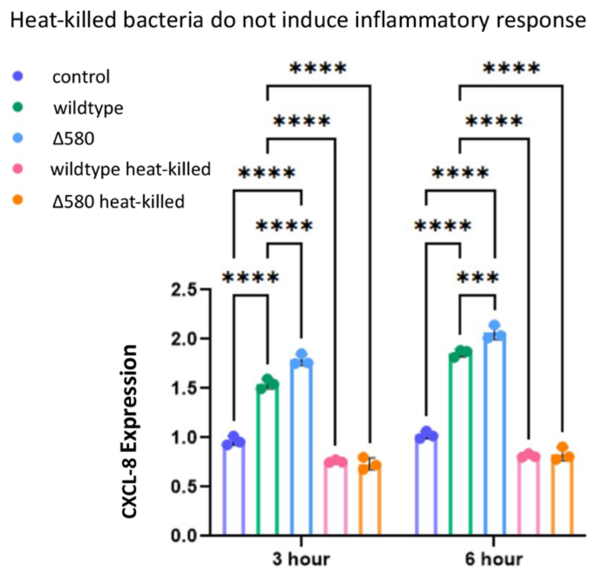

Figure S1. Heat-killed bacteria from wildtype and glycoprotein biosynthesis mutants (Δ579, Δ580) did not elicit CXCL-8 secretion from treated AGS cells relative to live cells. Heat-killed bacteria demonstrated significantly lower CXCL-8 secretion relative to their live counterparts following incubation with AGS cells. AGS-bacteria incubations were performed with live and heat-killed bacteria in parallel. Measurements were taken at 3- and 6-hour incubation time points, with negligible differences between the time-points. Heat-killed bacteria induced CXCL-8 levels comparable to the negative control that was not treated with bacteria. All statistics were calculated via a Tukey's multiple comparison test ANOVA. (* $P < 0.05$, ** $P < 0.01$, *** $P < 0.001$, **** $P < 0.0001$, ns = not significant)

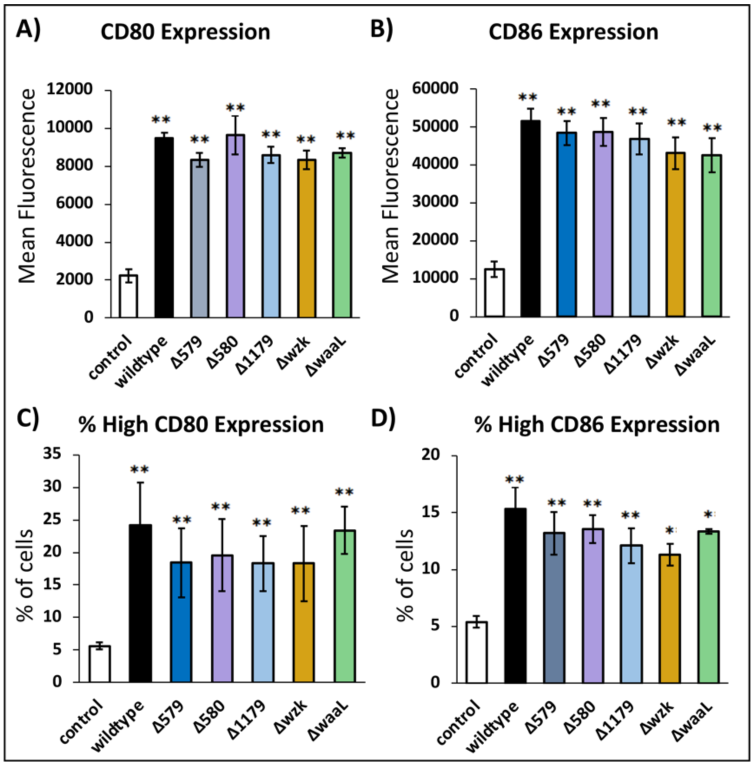

Figure S2. Immature dendritic cells (iDCs) were activated when challenged with wildtype *H. pylori* and glycosylation mutants. (A-B) Flow cytometry analysis revealed that iDCs expressed more (A) CD80 and (B) CD86 following exposure to wildtype *H. pylori* and glycosylation mutants (Δ579, Δ580, Δ1179, Δwzk, ΔwaaL) relative to iDCs treated with no bacteria (control) (**P < 0.01). There were no significant differences among the bacteria-treated samples (P > 0.05). Mean fluorescence intensity was calculated by averaging the mean fluorescence of three replicates, and error bars represent the standard deviation of replicates. (C-D) Percent of iDCs exhibiting high levels of (C) CD80 and (D) CD86 enhanced significantly following treatment with bacterial strains versus with no bacteria (* P < 0.05, ** P < 0.01). There were no significant differences in activation of iDCs challenged with wildtype *H. pylori* versus glycosylation mutants (P > 0.05). Data are representative of replicate experiments (n >3). All statistics were calculated via a Tukey's multiple comparison test ANOVA.

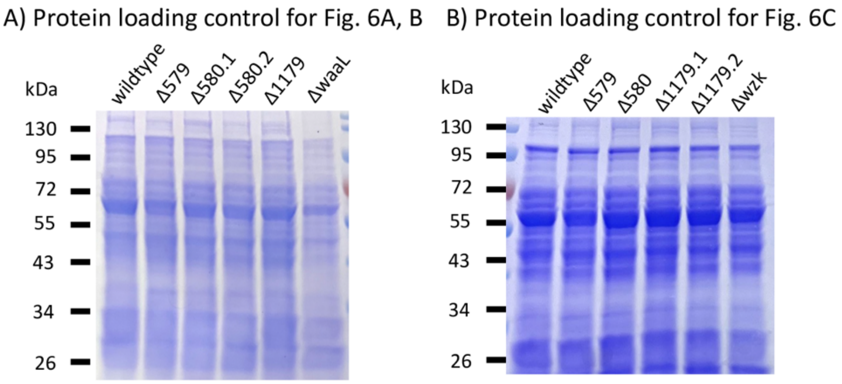

Figure S3. Protein loading controls for samples analyzed in Figure 6. A) Protein loading control for samples analyzed in Figure 6A and 6B to assess Lewis Y expression and LPS elaboration across *H. pylori* strains. Protein concentrations were standardized across samples, then proteins were electrophoresed and visualized by Coomassie staining. B) Protein loading control of samples from Figure 6C prior to digestion with proteinase K to prepare LPS. Proteins were electrophoresed and visualized by Coomassie staining.

4 Supplementary References

(1) Darling, A. C.; Mau, B.; Blattner, F. R.; Perna, N. T. Mauve: multiple alignment of conserved genomic sequence with rearrangements. *Genome Research* **2004**, *14* (7), 1394-1403.
(2) Delcher, A. L.; Bratke, K. A.; Powers, E. C.; Salzberg, S. L. Identifying bacterial genes and endosymbiont DNA with Glimmer. *Bioinformatics* **2007**, *23* (6), 673-679.
(3) Arkin, A. P.; Cottingham, R. W.; Henry, C. S.; Harris, N. L.; Stevens, R. L.; Maslov, S.; Dehal, P.; Ware, D.; Perez, F.; Canon, S.; et al. KBase: The United States Department of Energy Systems Biology Knowledgebase. *Nature Biotechnology* **2018**, *36* (7), 566-569.
(4) Aziz, R. K.; Bartels, D.; Best, A. A.; DeJongh, M.; Disz, T.; Edwards, R. A.; Formsma, K.; Gerdes, S.; Glass, E. M.; Kubal, M.; et al. The RAST Server: Rapid Annotations using Subsystems Technology. *BMC Genomics* **2008**, *9* (1), 75.
(5) Altschul, S. F.; Gish, W.; Miller, W.; Myers, E. W.; Lipman, D. J. Basic local alignment search tool. *Journal of Molecular Biology* **1990**, *215* (3), 403-410.
(6) Eddy, S. R. Accelerated Profile HMM Searches. *PLoS Comput Biol* **2011**, *7* (10), e1002195.
(7) El-Gebali, S.; Mistry, J.; Bateman, A.; Eddy, S. R.; Luciani, A.; Potter, S. C.; Qureshi, M.; Richardson, L. J.; Salazar, G. A.; Smart, A.; et al. The Pfam protein families database in 2019. *Nucleic Acids Research* **2019**, *47* (D1), D427-d432.
(8) Kelley, L. A.; Mezulis, S.; Yates, C. M.; Wass, M. N.; Sternberg, M. J. E. The Phyre2 web portal for protein modeling, prediction and analysis. *Nature Protocols* **2015**, *10* (6), 845-858.
(9) Lizak, C.; Gerber, S.; Numao, S.; Aebi, M.; Locher, K. P. X-ray structure of a bacterial oligosaccharyltransferase. *Nature* **2011**, *474* (7351), 350-355.
(10) Bai, L.; Kovach, A.; You, Q.; Kenny, A.; Li, H. Structure of the eukaryotic protein O-mannosyltransferase Pmt1-Pmt2 complex. *Nature Structural Molecular Biology* **2019**, *26* (8), 704-711.
(11) Gibson, D. G.; Young, L.; Chuang, R.-Y.; Venter, J. C.; Hutchison, C. A.; Smith, H. O. Enzymatic assembly of DNA molecules up to several hundred kilobases. *Nature Methods* **2009**, *6* (5), 343-345.
(12) Moulton, K. D.; Adewale, A. P.; Carol, H. A.; Mikami, S. A.; Dube, D. H. Metabolic Glycan Labeling-Based Screen to Identify Bacterial Glycosylation Genes. *ACS Infectious Diseases* **2020**. DOI: 10.1021/acsinfecdis.0c00612.
(13) Kiick, K. L.; Saxon, E.; Tirrell, D. A.; Bertozzi, C. R. Incorporation of azides into recombinant proteins for chemoselective modification by the Staudinger ligation. *Proceedings of the National Academy of Sciences* **2002**, *99* (1), 19.
(14) Koenigs, M. B.; Richardson, E. A.; Dube, D. H. Metabolic profiling of *Helicobacter pylori* glycosylation. *Molecular BioSystems* **2009**, *5* (9), 909-912, 10.1039/B902178G.
(15) Pohl, M. A.; Kienesberger, S.; Blaser, M. J. Novel Functions for Glycosyltransferases JHP0562 and GalT in Lewis Antigen Synthesis and Variation in *Helicobacter pylori*. *Infection and Immunity* **2012**, *80* (4), 1593-1605.
(16) Hug, I.; Couturier, M. R.; Rooker, M. M.; Taylor, D. E.; Stein, M.; Feldman, M. F. *Helicobacter pylori* lipopolysaccharide is synthesized via a novel pathway with an evolutionary connection to protein N-glycosylation. *PLoS Pathogens* **2010**, *6* (3), e1000819.
(17) Li, H.; Marceau, M.; Yang, T.; Liao, T.; Tang, X.; Hu, R.; Xie, Y.; Tang, H.; Tay, A.; Shi, Y.; et al. East-Asian *Helicobacter pylori* strains synthesize heptan-deficient lipopolysaccharide. *PLOS Genetics* **2019**, *15* (11), e1008497.

(18) Baltrus, D. A.; Amieva, M. R.; Covacci, A.; Lowe, T. M.; Merrell, D. S.; Ottemann, K. M.; Stein, M.; Salama, N. R.; Guillemin, K. The complete genome sequence of *Helicobacter pylori* strain G27. *Journal of Bacteriology* **2009**, *191* (1), 447-448.
(19) Yang, D. C.; Blair, K. M.; Taylor, J. A.; Petersen, T. W.; Sessler, T.; Tull, C. M.; Leverich, C. K.; Collar, A. L.; Wyckoff, T. J.; Biboy, J.; et al. A genome-wide *Helicobacter pylori* morphology screen uncovers a membrane-spanning helical cell shape complex. *Journal of Bacteriology* **2019**, *201* (14).
